# The Parvalbumin Hypothesis of Autism Spectrum Disorder

**DOI:** 10.3389/fncel.2020.577525

**Published:** 2020-12-18

**Authors:** Federica Filice, Lucia Janickova, Thomas Henzi, Alessandro Bilella, Beat Schwaller

**Affiliations:** Section of Medicine, Anatomy, University of Fribourg, Fribourg, Switzerland

**Keywords:** parvalbumin, autism (ASD), calcium signal modulator, GABAergic neurons, E/I balance, schizophrenia, ROS, mitochondria

## Abstract

The prevalence of autism spectrum disorder (ASD)—a type of neurodevelopmental disorder—is increasing and is around 2% in North America, Asia, and Europe. Besides the known genetic link, environmental, epigenetic, and metabolic factors have been implicated in ASD etiology. Although highly heterogeneous at the behavioral level, ASD comprises a set of core symptoms including impaired communication and social interaction skills as well as stereotyped and repetitive behaviors. This has led to the suggestion that a large part of the ASD phenotype is caused by changes in a few and common set of signaling pathways, the identification of which is a fundamental aim of autism research. Using advanced bioinformatics tools and the abundantly available genetic data, it is possible to classify the large number of ASD-associated genes according to cellular function and pathways. Cellular processes known to be impaired in ASD include gene regulation, synaptic transmission affecting the excitation/inhibition balance, neuronal Ca^2+^ signaling, development of short-/long-range connectivity (circuits and networks), and mitochondrial function. Such alterations often occur during early postnatal neurodevelopment. Among the neurons most affected in ASD as well as in schizophrenia are those expressing the Ca^2+^-binding protein parvalbumin (PV). These mainly inhibitory interneurons present in many different brain regions in humans and rodents are characterized by rapid, non-adaptive firing and have a high energy requirement. PV expression is often reduced at both messenger RNA (mRNA) and protein levels in human ASD brain samples and mouse ASD (and schizophrenia) models. Although the human *PVALB* gene is not a high-ranking susceptibility/risk gene for either disorder and is currently only listed in the SFARI Gene Archive, we propose and present supporting evidence for the Parvalbumin Hypothesis, which posits that decreased PV level is causally related to the etiology of ASD (and possibly schizophrenia).

## Introduction

Complex mechanisms underlie neurodevelopmental disorders (NDDs) and neuropsychiatric conditions. From the molecular to the system level, subtle changes at any point during development can lead to impairment of brain functions including cognition, learning, memory, and behavior, which are dynamic and subject to developmental and environmental influences and activity/experience-dependent mechanisms. Genome-wide association studies, investigation of rare genetic disorders, and transcriptome analyses aim to identify genes implicated in the etiology of NDDs, and bioinformatics approaches are used to elucidate dysregulated processes/pathways. The interplay between altered genes, circuits, and networks giving rise to clinical symptoms in NDDs is illustrated in Figure 1 of Krol et al. (2018).

The motivation for the present article comes from many studies on ASD and schizophrenia focusing on the calcium-binding protein parvalbumin (PV) expressed by a subpopulation of neurons (sometimes referred to as fast-spiking interneurons (FSIs) or PV^+^ interneurons; Hu et al., 2014). There is some confusion in the literature regarding the nomenclature. In most morphological studies using immunohistochemical methods, PV^+^ neurons are defined as those labeled by anti-PV antibody. A case where the number of PV-immunoreactive neurons is lower than in control animals as a result of experimental treatment or genetic manipulation is either correctly described as a reduction in the number of PV^+^ neurons or otherwise as a loss of PV^+^ neurons. The latter implies that these neurons either died or did not properly migrate to a specific brain region during development as a result of the manipulation, while a third possibility is that PV was downregulated to a level below the limit of detection by immunohistochemistry (IHC). Thus, distinguishing between PV^+^ neuron loss vs. decreased PV expression is critical for accurately interpreting previous studies. For the remainder of this article, we use the term PV^+^ neurons only in reference to neurons identified solely by PV IHC; the class of interneurons defined by PV expression as well as additional features (e.g., morphology, electrophysiological properties, and transcriptome profile) is referred to as Pvalb neurons irrespective of PV expression level. As an example, brains of PV^−/−^ mice completely lacking PV expression were found to harbor the same number of Pvalb neurons as wild-type (WT) mice (Filice et al., 2016).

**Figure 1:**
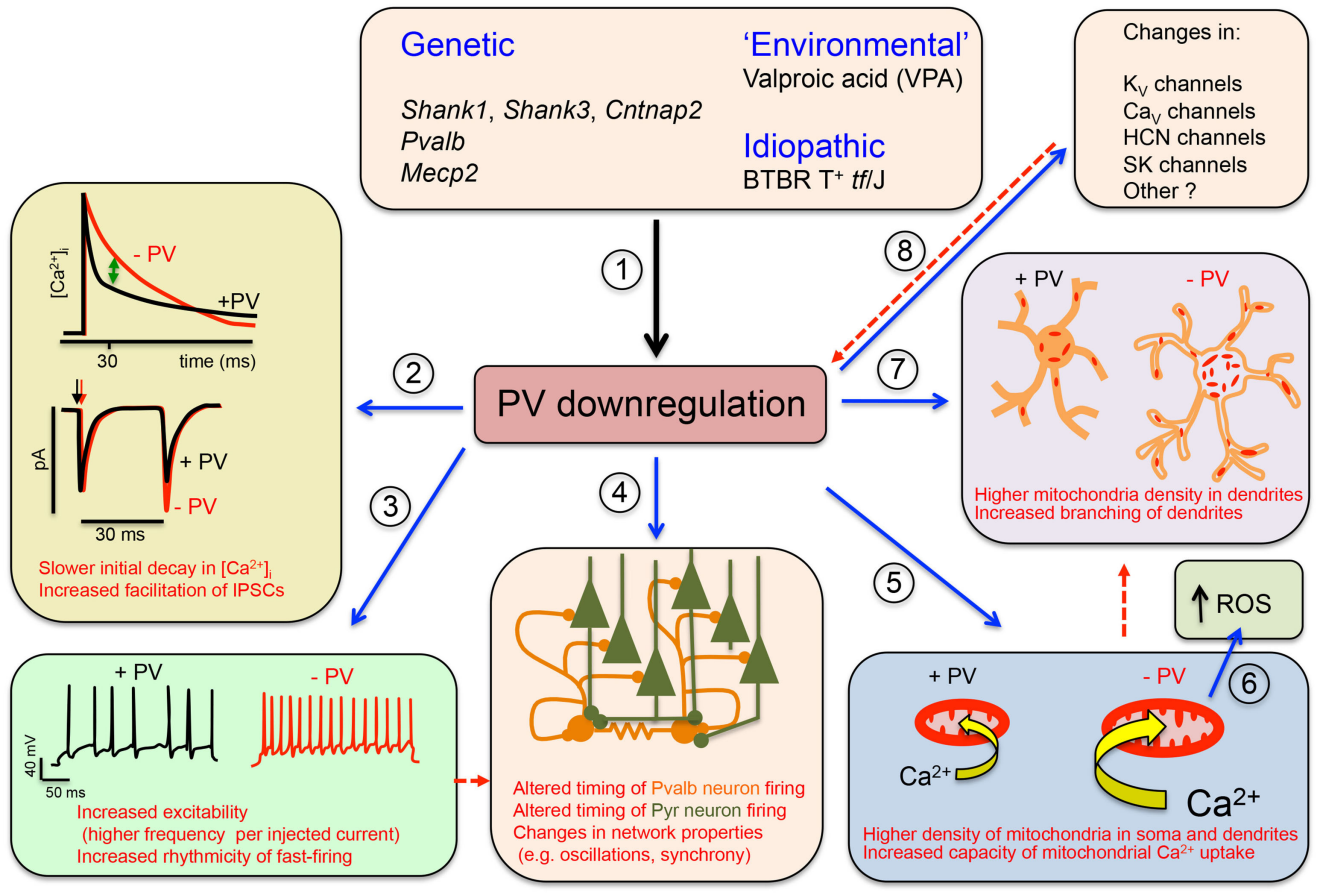
Graphical summary of the Parvalbumin Hypothesis of autism spectrum disorder (ASD) (and putatively, of schizophrenia). (1) Genetic and environmental factors directly or indirectly affecting Pvalb neuron function in mice through impairment of principal neurons, leading to the downregulation of parvalbumin (PV) and reduced neuronal function. (2) (Upper) The initial decline in [Ca^2+^]_i_ in Pvalb neurons is accelerated in the presence of PV. Differences in the decay curves (±PV) are largest in the time window of ~20–50 ms after peak [Ca^2+^]_i_, resulting in increased residual Ca^2+^ in the absence of PV (green arrow). (Lower) Larger amounts of residual Ca^2+^ promote γ-aminobutyric acid (GABA) release during further stimulation in this time window, leading to increased facilitation in the paired-pulse protocol. (3) An absence of PV alters other electrophysiological properties: for example, it may increase the excitability of Pvalb neurons, or may result in more regular firing within AP trains (i.e., reducing “jitter”) (modified from Orduz et al., 2013). (4) A decrease in PV level affects the electrophysiological properties not only of Pvalb neurons but also of principal (pyramidal) cells and modifies network properties such as oscillation and synchrony. (5) A decrease in PV level alters Ca^2+^-dependent excitation/transcription coupling by reducing transcript levels of activity-driven genes including *Pvalb* and *Gad1* and prototypical neuronal-activity-associated genes such as *c-Fos, Arc*, and *Wnt2*; it also increases messenger RNA (mRNA) levels of genes involved in mitochondrial biogenesis such as *Ppargc1a* encoding the mitochondria master regulator PGC-1α, as well as *Nrf1* and *Tfam*. This results in an increase in mitochondrial volume (density) that is approximately proportional to the PV concentration in corresponding wild-type (WT) Pvalb neurons—i.e., the higher the PV concentration, the greater the increase in mitochondrial density in PV^−/−^ Pvalb neurons. (6) Increased mitochondrial volume associated with a decrease in PV level enhances mitochondrial Ca^2+^-buffering/sequestration capacity, thereby promoting reactive oxygen species (ROS) production. This slow process may impair mitochondrial function and ultimately impacts Pvalb neuron function, which is thought to produce a schizophrenia phenotype. (7) Absence of PV during (early) postnatal neurodevelopment from ~PND7 to PND20 increases dendritic branching, yielding Pvalb neurons with a larger dendritic tree and possibly resulting in hyperconnectivity. Increases in mitochondria number and dendritic branching caused by PV downregulation may be subsequent events (red dashed arrow) or could occur via independent mechanisms. (8) Absence of PV perturbs the expression of other putative ASD susceptibility genes that are either directly associated with the electrophysiological properties of Pvalb neurons or are expressed in pyramidal cells that influence Pvalb neuron circuitry; this includes K_V_, Ca_V_ HCN channels, and SK channels. Whether targeted inhibition of these genes also affects PV level is unknown. Solid arrows indicate a causal relationship between events—e.g., altered Ca^2+^ concentration (±PV) and short-term modulation of synaptic plasticity. Dashed lines indicate putative (indirect) mechanism(s) via as yet unknown pathways. Points (1)–(8) are described in detail in the main text (see all subsections in *Data Supporting the Parvalbumin Hypothesis of ASD*).

The Parvalbumin Hypothesis of ASD was formulated based on the following lines of evidence: (1) the function of Pvalb neurons is often impaired in NDDs and neuropsychiatric disorders (Marin, 2012; Ferguson and Gao, 2018; Selten et al., 2018); (2) *PVALB* mRNA is the most strongly downregulated transcript in human ASD samples (Schwede et al., 2018); (3) the number of PV^+^ neurons is decreased in sections of human ASD brain (Hashemi et al., 2017; Ariza et al., 2018); and (4) transgenic mice with lower (PV^+/−^) or absent (PV^−/−^) expression exhibit the core ASD-like symptoms (Wöhr et al., 2015); the main points of the Parvalbumin Hypothesis are graphically summarized in Figure 1.

## Data Supporting the Parvalbumin Hypothesis of ASD

### Decreased Number of PV^+^ Neurons in Human ASD Postmortem Brains: Pvalb Neuron Loss vs. PV Downregulation

The cerebral cortex harbors several classes of interneurons including Pvalb neurons. Early classification of these neurons was based on their morphology [most notably of the axon (Kawaguchi and Kubota, 1997; Toledo-Rodriguez et al., 2005)], firing pattern, PV expression, and gene expression profile determined by PCR (Toledo-Rodriguez et al., 2004). More recent classifications based on single-cell or single-nucleus RNA-sequencing (RNA-seq) (Hodge et al., 2019), patch sequencing (Patch-seq) yielding morphoelectric (*met*-type) and transcriptomic (*t*-type) datasets (Gouwens et al., 2020), or machine learning applied to a large patch-seq dataset (Gala et al., 2020) have identified 7 clusters (RNA-seq) as well as 10 *t*-type and 5 *met*-type (patch-seq) Pvalb neurons. Given the different algorithms used to stratify the datasets, it is not surprising that the reported number of Pvalb neuron subtypes varies across studies. Pvalb neurons in the cortex include several types of basket cell, chandelier cell, and translaminar neurons. Basket cells localized in layers 2–6 constitute the majority; chandelier cells are abundant at the boundary between layers 1 and 2 and in layer 6; and the relatively rare translaminar Pvalb neurons are mostly present in layers 5 and 6 (Lim et al., 2018). A similar classification has been applied to hippocampal Pvalb neurons (Somogyi and Klausberger, 2005).

In order to conclude that PV^+^ neurons are absent in postmortem ASD brains, it must be demonstrated that neurons with specific morphoelectric, transcriptomic, and biochemical properties are missing/reduced. The typically fixed postmortem human brain tissue samples preclude the assessment of these properties. Besides non-adaptive firing, PV^+^ neurons are characterized by PV expression. However, the complete absence of PV cannot be taken as evidence that Pvalb neurons are absent, which must be determined based on the expression of other Pvalb neuron-specific markers including voltage-gated potassium channel K_V_3.1b (*KCNC1*), potassium voltage-gated channel modifier subfamily S member 3 K_V_9.3 (*KCNS3*), or components of perineuronal nets (PNNs), which are a set of extracellular matrix molecules surrounding Pvalb neurons in several brain regions including the cortex (Celio et al., 1998). The presence and/or intensity of PNN immunolabeling must be interpreted with caution because PNN components, like other Pvalb neuron markers besides PV (*PVALB*)—e.g., GAD67 (*GAD1*)—are regulated in an experience/activity-dependent manner (Cohen et al., 2016), and their expression changes over the course of development (Wang and Fawcett, 2012; Ye and Miao, 2013). Earlier studies found no significant changes in the number of PV^+^ or calbindin-D28K-positive (CB^+^) neurons in the posterior cingulate cortex and fusiform gyrus of postmortem ASD brains (Oblak et al., 2011). However, the mean density of PV^+^ neurons was slightly reduced in the superficial and deep layers of both of these areas. In another study, the density of PV^+^ cerebellar stellate and basket cells did not differ significantly between ASD (*n* = 6) and control (*n* = 4) samples, although the number of Purkinje cells was reduced in two or three ASD samples (Whitney et al., 2009). Moreover, the mean neuron density of both interneuron subtypes was reduced (basket cells, −15%; stellate cells, −5%), although the difference was not statistically significant. These findings imply that the number of PV^+^ neurons is decreased or PV is downregulated in ASD. In a preliminary study (Zikopoulos and Barbas, 2013) comparing the density of CB^+^ and PV^+^ cortical interneurons in postmortem adult human brain tissue (ASD and control; *n* = 2) from dorsolateral prefrontal area 9, a significant reduction in the number of PV^+^ neurons was observed in ASD brains; the ratio of PV^+^/CB^+^ interneurons was 0.65 as compared to ~1 in controls. The lower ratio was assumed to reflect a decreased number of PV^+^ interneurons (Zikopoulos and Barbas, 2013). An analysis of cortical interneurons expressing calretinin (CR), CB, and PV in postmortem brains of ASD and age-matched control cases revealed significant differences in three regions of the prefrontal cortex (PFC) (i.e., BA9, BA46, and BA47) that have been implicated in ASD (Hashemi et al., 2017): compared to control cases, the proportion of PV^+^ neurons was decreased by 70% in BA46, 45% in BA9, and 38% in BA47 in ASD subjects, while the proportion of neurons expressing CB or CR did not differ between the two groups. Reduced numbers of PV^+^ neurons were equally found in the supra- and infragranular layers. The authors speculated that the decrease in PV^+^ neurons was due to the loss of Pvalb neurons or downregulation of PV protein expression. In a follow-up study (Ariza et al., 2018), PV^+^ interneuron subtypes (basket and chandelier cells) were separately analyzed in the same samples (Hashemi et al., 2018); double immunolabeling of PV and PNNs with *Vicia villosa agglutinin* (VVA) enabled the differentiation of basket and chandelier cells, as only the former are surrounded by VVA^+^ PNNs in human brain. The number of PV^+^ chandelier cells was consistently lower in the PFC of ASD specimens (*n* = 10); the number of PV^+^/VVA^+^ basket cells was also reduced but to a lesser degree. As the authors did not use a second marker to unambiguously identify chandelier cells, they concluded that the reduction in the number of PV^+^ cells represents either a true loss of Pvalb neurons (mostly chandelier cells) or a decrease in PV protein level.

PV expression is developmentally regulated in humans and rodents. It has been suggested that gene expression changes in NDDs are related to an immature developmental gene expression program of PV^+^ FSIs (Gandal et al., 2012). Cell-type-specific maturation indices calculated from microarray datasets showed that the index for Pvalb neurons was highly correlated with PV expression level in these cells. In all three analyzed diseases (ASD, schizophrenia, and bipolar disorder), the maturation index and PV expression level were significantly reduced, consistent with a delayed/impaired developmental gene expression program. While there are currently no quantitative data on PV expression in human ASD brains, *PVALB* mRNA level has been measured in several studies. Radioisotopic *in situ* hybridization in postmortem brains of ASD and control cases revealed that *PVALB* transcript level was downregulated in cerebellar Purkinje cells of ASD brains, independent of postmortem interval or age at death (Soghomonian et al., 2017). An examination of neocortical architecture in postmortem brain specimens of autistic and normal children aged between 2 and 15 years revealed discrete patches of abnormal and disorganized laminar cytoarchitecture in most samples (Stoner et al., 2014). An analysis of specific molecular markers by RNA *in situ* hybridization showed abnormal gene expression in these neocortical regions: the level of the interneuron marker *PVALB* was decreased, whereas no difference was observed in the total number of neurons by Nissl staining. In accordance with the *in situ* hybridization data, analyses of an RNA-seq dataset (Parikshak et al., 2016) and three microarray datasets (Garbett et al., 2008; Voineagu et al., 2011; Chow et al., 2012) identified *PVALB* mRNA as the most strongly downregulated transcript in the cerebral cortex of ASD patients (Schwede et al., 2018).

The Ca^2+^/CaM-dependent protein kinase (CaMK) pathway is implicated in Ca^2+^/activity-dependent gene expression (Ebert and Greenberg, 2013), including in Pvalb neurons (Cohen et al., 2016). Many neurons are regulated by CaMK II and/or IV (reviewed in Bayer and Schulman, 2019). However, Pvalb neurons use the less common isoform γCAMK I (*Camk1g*) to induce cAMP response element-binding protein (CREB) phosphorylation and expression of prototypical activity-driven genes (*c-Fos, Arc*, and *Wnt2*) as well as *Gad1* and *Pvalb* (Cohen et al., 2016). The observation that the *CAMK1G* transcript is among the top 40 (out of >250) significantly downregulated mRNAs detected in combined gene array datasets of human ASD samples (Schwede et al., 2018) suggests that impaired activity of Pvalb neurons in ASD results in (likely γCAMK I-dependent) reduced PV expression. Additionally, coordinated dysregulation of genes implicated in synaptic and mitochondrial functions was observed in ASD specimens (Schwede et al., 2018). The possible link between *PVALB* dysregulation and mitochondria is discussed in greater detail in *Effect of PV on Mitochondria, Oxidative Stress, and Pvalb Neuron Morphology: Link to NDDs*.

In summary, *PVALB* transcript level is not only reduced in the brain of ASD cases compared to normal controls but also shows the strongest downregulation among differentially expressed genes. Regarding Pvalb neuron loss, no study to date has provided definitive evidence (using Pvalb neuron markers such as K_V_3.1b or K_V_9.3) of a lower number/density of Pvalb neurons in human ASD brains. Even if this was demonstrated, an outstanding question would be whether this is due to the death of true Pvalb neurons (i.e., Pvalb neuron-selective neurodegeneration), defects in migration or maturation of immature Pvalb neurons during development (e.g., as seen in Dlx5/6^−/−^ mice; Wang et al., 2010), or another mechanism. The downregulation of PV may be linked to loss of Pvalb neuron function, although a partial decrease in function resulting from the abovementioned processes cannot be excluded.

### Animal NDD Models of ASD With Reduced PV Expression and/or Decreased Number/Altered Distribution of PV^+^ Neurons

Various animal models have been used to investigate NDDs including ASD. ASD risk genes are defined as those that are more frequently mutated in ASD patients than is expected by chance. Large-scale exome sequencing has identified 102 such genes (Satterstrom et al., 2020). An animal (mouse) model harboring the same (or similar) mutations and shows ASD-like behavior has high construct validity. However, in recent years, numerous mouse models (inbred, experimentally induced, and transgenic) have been discovered or established that show varying degrees of typical ASD-like core symptoms. A list of such models (>1,000) is available in the SFARI Gene database (https://gene.sfari.org/), with genes putatively associated with ASD ranked according to several criteria. More models (>3,000) are listed in the AutDB database (http://autism.mindspec.org/autdb/Welcome.do). The number of (mostly mouse) ASD models is constantly increasing, with the models themselves becoming more sophisticated as a result of the possibility of inactivating/deleting genes in a brain region- and neuron type-specific and temporally regulated manner. The last aspect is particularly important for elucidating the etiology of NDDs. Although many mouse models currently used in basic research lack robust construct validity, their strong face validity [anatomic, biochemical, neuropathological, or behavioral phenotype(s) similar to humans] make them highly useful for mechanistic studies on ASD etiology. In this paper, we focus on NDD models affecting Pvalb neurons in terms of neuron number and function and PV expression level, irrespective whether they are currently classified as models of validated (syndromic) ASD risk genes.

The functional impairment of Pvalb neurons has attracted considerable attention in ASD research (Gogolla et al., 2009; Marin, 2012). The distribution of Pvalb neurons in the rat nervous system has been described before (Celio, 1990). A more detailed and quantitative analysis of Pvalb neuron distribution was carried out using mice in which green fluorescent protein (GFP) fused to histone 2B was selectively expressed in Pvalb neurons (PV-Cre:H2B-GFP) (Kim et al., 2017). In the same study, the distribution of other interneuron classes [somatostatin-positive (SST^+^) and vasoactive intestinal polypeptide-positive (VIP^+^) interneurons] was analyzed in parallel; the results showed an uneven distribution of the three major interneuron types in the isocortex. The density of the different interneuron subtypes varies in different cortical layers (Kim et al., 2017). Known changes in PV expression (mainly determined by IHC) or in Pvalb neuron function (detected by electrophysiological recordings) are summarized in Table 1. In many mouse models, PV IHC has revealed an overall reduction in PV^+^ cell numbers in brain regions associated with ASD including the somatosensory cortex (SSC), medial (m)PFC, striatum, hippocampus, thalamic reticular nucleus (TRN), and cerebellum. Importantly, the choice of brain region for investigating PV expression has been somewhat arbitrary and likely dictated by other types of experiments (usually electrophysiology). Thus, if a significant difference in PV expression is observed in a particular brain region, it cannot be assumed that differences also exist in other brain regions. Therefore, systematic analyses are warranted. In some cases, changes in the number/distribution of PV^+^ cells in a particular brain region are correlated with altered electrophysiological properties. For example, in Ambra1^+/−^ mice, a likely reduction in PV—observed as a decrease in PV^+^ cell number and PV protein level and interpreted as a loss of hippocampal PV interneurons (Nobili et al., 2018)—was correlated with an overall reduction in inhibitory input from Pvalb neurons in CA1 pyramidal cells. Moreover, the perisomatic paired-pulse ratio (PPR) was increased in female Ambra1^+/−^ mice, which was also consistent with PV downregulation. These changes lead to hyperexcitability of CA1 pyramidal neurons and excitatory/inhibitory (E/I) imbalance (Table 1). Of note, similar findings were observed in Ehmt1^+/−^ mice—a model for delayed circuit maturation—evidenced by a reduced number of PV^+^ neurons at early age (PND14) in several cortical regions and characterized by impaired E/I balance (Table 1).

**Table 1:** Mouse models with evidence of parvalbumin (PV) alterations and associated changes in electrophysiology and occurrence of autism spectrum disorder (ASD)-like behavior.

**Mouse model/*Gene name*/Protein name SFARI classification**	**Type of PV alteration(s)/age of testing**	**Electrophysiological alteration(s) relevant to Pvalb neuron function**	**ASD-like behavioral impairments (core and comorbidity)**
**(A) GENETIC MODELS LISTED IN THE SFARI GENE DATABASE (https://gene.sfari.org/) (S, SYNDROMIC)**			
**Cntnap2**^**−/−**^ ***Cntnap2*** Contactin associated protein-like 2 SFARI 2 (S)	**STRIATUM, CORTEX** (Penagarikano et al., 2011;Vogt et al., 2018) Reduction in the number of PV^+^ cells (P14 and P30) **STRIATUM** (Lauber et al., 2018) Reduction in the number of PV^+^ cells and *Pvalb* mRNA, **no change** in the number of VVA^+^ (Pvalb) neurons (P25)	**HIPPOCAMPUS** (Jurgensen and Castillo, 2015) **Altered inhibition onto CA1 pyramidal neurons** • Reduced amplitude of putative perisomatic-evoked IPSCs • Increase in the frequency of spontaneous AP-driven IPSCs **SSC** (Antoine et al., 2019) **Reduced inhibition and (feebly) decreased excitation in L2/L3 pyramidal cells** • Impairment in Pvalb neuron-mediated feedforward eIPSCs • Reduced spontaneous spiking frequency of mEPSCs and mIPSCs	Stereotypic motor movements and communication and social abnormalities. Hyperactivity to thermal sensory stimuli (Penagarikano et al., 2011)
**Ehmt1**^**+/−**^ ***Ehmt1*** Euchromatic histone Methyltransferase 1 SFARI Gene Archive 3 (S)	**AUDITORY CORTEX (AC) L2/3 and 4, SSC L2/3 and 4, VISUAL CORTEX (VC) L2/3 and 4** (Negwer et al., 2020) Delay of PV^+^ neuron maturation in early (P14) sensory development, with layer- and region-specific variability later (P28, P56) **AC**: Decrease in PV^+^ neurons in **L2/3 and 4** at P14 only **SSC**: reduced number of PV^+^/VVA^+^ neurons at all ages in **L4** only **VC**: no differences in **L2/3** at all ages; reduced number of PV^+^ and PV^+^/VVA^+^ in **L4** at P14	**HIPPOCAMPAL ACUTE SLICES** (Frega et al., 2020) **Impairment in inhibitory transmission** • Strong reduction of mIPSC amplitude and frequency in CA1 pyramidal cells at P21 • Increased inhibitory PPR responses specifically at 50 ms ISI in CA1 pyramidal cells following stimulation in *stratum radiatum* (P21) • Hyperexcitability in CA1 hippocampal excitatory neurons • Results indicate that Ehmt1 plays a role in controlling E/I balance by regulating inhibitory inputs onto CA1 pyramidal cells **AUDITORY CORTEX ACUTE SLICES** (Negwer et al., 2020) • Reduced mIPSC frequency, NOT amplitude in auditory cortex L2/3 pyramidal cells at P14–16. • Increased inhibitory PPR responses at ISI of 50, 100, and 200 ms • Decreased release probability of GABA determined from a 10-Hz stimulus train	Reduced social play in juvenile (P30) male, but not in female mice Prolonged social approach in the social approach assay (3-chamber assay) observed in adult (3 months) male and female Ehmt1^+/−^ mice Delayed (males) or absent (females) preference for social novelty Hypergrooming and reduced exploration in the open field (Balemans et al., 2010)
**En2**^**−/−**^ ***En2*** Engrailed 2 SFARI 3	**HIPPOCAMPUS, SSC** (Tripathi et al., 2009) Reduction in the number of PV^+^ cells (3–5 months) **TRN** (Provenzano et al., 2020) Reduction of *Pvalb* mRNA (P30) Reduction in the number of PV^+^ cells (6 weeks) **VISUAL CORTEX (L2/3 and L5/6)** (Pirone et al., 2020) Increase in the number of PV^+^ cells (P30 and adult)	No electrophysiology at cellular level reported	Deficits in reciprocal social interactions as juveniles and adults (Brielmaier et al., 2012)
**Fmr1**^**−/−**^ ***Fmr1*** Fragile X-mental retardation protein (FMRP) SFARI 3 (S)	**SSC** (Selby et al., 2007) Reduction in the number of PV^+^ cells (12 months) **DEVELOPING AUDITORY CORTEX** (Wen et al., 2019) Reduction in the number of PV^+^ cells (P14, P21, P30)	**SSC (barrel cortex layer 4)** (Gibson et al., 2008) **Reduced feedback inhibition mediated by FS (Pvalb) neurons** • Decreased EPSC frequency on FS (Pvalb) neurons • Normal feed-forward inhibition > no change in inhibitory drive • Excitatory neurons intrinsically more excitable > increased evoked AP firing rate during 600-ms current injection	ASD-like core symptoms of altered social interaction and repetitive behaviors, hyperactivity (Pietropaolo et al., 2011). Mental retardation, anxiety, increased incidence of epilepsy, auditory hypersensitivity (Chen and Toth, 2001;Frankland et al., 2004)
**(A) GENETIC MODELS LISTED IN THE SFARI GENE DATABASE (https://gene.sfari.org/) (S, SYNDROMIC)**			
**Mecp2**^**−/−**^ ***Mecp2*** Methyl-CpG-binding Protein-2 SFARI 2 (S)	**VISUAL CORTEX (V1)** (Krishnan et al., 2015;Patrizi et al., 2019) Accelerated maturation of PV network (P14 and P30) **HIPPOCAMPUS CA3** (Calfa et al., 2015) Density of PV^+^ interneurons not altered in CA3 (P40–P60) **VISUAL CORTEX** (Durand et al., 2012) Increased *Pvalb* mRNA levels PV-hyperconnectivity > increased PV immunofluorescence intensity as consequence of increased neurite complexity (from P15 on; persistent also in adulthood)	**HIPPOCAMPUS CA3** (Calfa et al., 2015) **Weaker synaptic inhibition onto CA3 pyramidal neurons resulting in larger E/I ratio in CA3 pyramidal neurons** • Smaller amplitude, but higher mIPSCs frequency in pyramidal neurons; larger amplitude, but lower frequency of mEPSCs • Smaller slope of input/output (I/O) relationship • Lower frequency of spontaneous APs from FS (Pvalb) neurons > smaller and less frequent mEPSCs onto CA3 fast-spiking basket cells **VISUAL CORTEX** (Durand et al., 2012) **Reduction of pyramidal neuron and network activity** • Stronger inhibition of pyramidal neurons caused by a hyperinnervation by PV-expressing interneurons > low spontaneous and evoked neuronal activity and a general silencing of cortical circuitry	Ataxia, stereotyped behaviors, seizures, motor, sensory, memory, and social deficits (Chao et al., 2010;Ito-Ishida et al., 2015)
**Nlgn3**^**−/−**^ ***Nlgn3*** Neuroligin 3 SFARI 2	**SSC** (Gogolla et al., 2009) “Patchy” PV^+^ cells (2–3 months)	**HIPPOCAMPUS CA2** (Modi et al., 2019) **Increased neuronal excitability and reduced inhibition** • Increased frequency of spontaneous EPSCs and decrease in frequency of spontaneous IPSCs in CA2 pyramidal cells • strong reduction of perisomatic inhibition mediated by CCK neurons **HIPPOCAMPUS CA1** (Polepalli et al., 2017) **Decrease in Pvalb interneuron inhibition** • Significant reduction in facilitation of EPSCs and spiking in Pvalb neurons lacking Nlgn3	Impaired social novelty and social memory, hyperactivity, repetitive behaviors (Tabuchi et al., 2007;Rothwell et al., 2014)
**Nlgn3 R451C** Knock-in substitution of Arg^451^ to Cys ***Nlgn3*** Neuroligin 3 SFARI 2	**SSC** (Speed et al., 2015) PV^+^ cell number unchanged (P13–17)	**SSC (barrel cortex)** (Tabuchi et al., 2007;Speed et al., 2015) **Increase in inhibitory synaptic transmission** • Increase in spontaneous mIPSC frequency in L2/3 pyramidal neurons • Increased amplitude of evoked IPSCs in L2/3 pyramidal neurons **Pvalb neuron–pyramidal cell synaptic connections NOT altered** • Unitary IPSC_5_/IPSC_1_ ratio unchanged; paired whole-cell recordings between Pvalb neurons and pyramidal neurons **HIPPOCAMPUS CA1** (Etherton et al., 2011) **Increase in AMPA and NMDA receptor-mediated excitatory synaptic transmission** • Increased fEPSP slope and spontaneous mEPSCs frequency **Increased evoked synaptic strength at inhibitory synapses** • Larger eIPSC amplitude in L2/3 pyramidal neurons **mPFC** (*in vivo* recordings) (Cao et al., 2018) **Decreased excitability of FS interneurons and dysfunction of gamma oscillation** • Reduced firing frequency recorded from FS interneurons	Impaired social interaction behaviors and enhanced spatial learning (Tabuchi et al., 2007;Etherton et al., 2011)
**(A) GENETIC MODELS LISTED IN THE SFARI GENE DATABASE (https://gene.sfari.org/) (S, SYNDROMIC)**			
**Shank3**^**−/−**^ ***Shank3*** SH3 and multiple ankyrin repeat domains 3 SFARI 1 (S)	**STRIATUM** (Filice et al., 2016) Reduction in the number of PV^+^ cells and *Pvalb* mRNA, **no change** in the number of VVA^+^ (Pvalb) neurons (P25) **INSULAR CORTEX** (Gogolla et al., 2014) Diminished PV^+^ puncta on pyramidal cells (P70–100)	**CORTICOSTRIATAL ACUTE SLICES** (Peca et al., 2011) **Disruption of striatal glutamatergic signaling** • Decreased corticostriatal population spike amplitude • Reduced frequency and amplitude of AMPA-mediated mEPSC in MSN **SSC** (Chen et al., 2020) **Excitatory neuron hyperactivity and inhibitory neuron hypoactivity** • *in vivo* population calcium imaging (basal recordings and after stimulation: vibrissae motion detection task	Hypergrooming, social deficits, sensory hyperactivity (Peca et al., 2011;Chen et al., 2020)
**(B) ‘ENVIRONMENTAL' AND IDIOPATHIC MODELS**			
**VPA** Environmental model of ASD (*in utero* exposure to valproic acid	**SSC** (Gogolla et al., 2014) “Patchy” distribution of PV^+^ cells (2–3 months) **SSC, STRIATUM** (Lauber et al., 2016) Reduction in the number of PV^+^ cells and *Pvalb* mRNA, **no change** in VVA^+^ (Pvalb) neurons (P25)	**TEMPORAL CORTEX** (Banerjee et al., 2013) **mIPSC impairment** • Reduced mIPSC frequency, but increased rise time and decay time in L2/3 pyramidal cells • Lower input/output response of evoked IPSCs	Reduced communication, social deficits, repetitive behavior, stimuli hypersensitivity (Schneider and Przewlocki, 2005;Markram et al., 2008;Gandal et al., 2010)
**BTBR T**^**+**^ **Itpr3**^**tf**^**/J (BTBR T**^**+**^ ***tf*****/J) strain** Idiopathic model of ASD This strain carries the mutations “a^**t**^” (non-agouti; black and tan) “*Itpr3^*tf*^*” (inositol 1,4,5-triphosphate receptor 3; tufted), and “T” (brachyury)	**INSULAR CORTEX** (Gogolla et al., 2014) Diminished PV^+^ puncta on pyramidal cells (P70–100) **ANTERIOR CINGULATE CORTEX** (Stephenson et al., 2011) Reduction in PV^+^ cells (8–10 weeks)	**INSULAR CORTEX** (Gogolla et al., 2014) **Impaired multisensory integration—weakened inhibitory circuitry** • Decreased mIPSCs frequency recorded in pyramidal cells	Social deficits, impairments in vocal communication, stereotypic, repetitive behaviors, exaggerated auditory responses at low-moderate tones (McFarlane et al., 2008;Scattoni et al., 2011;Gogolla et al., 2014)
**(C) GENETIC NDD MODELS WITH A FEEBLER LINK TO PV AND ASD**			
**Ambra1**^**+/−**^ **(female mice)** ***Ambra1*** Activating molecule in Beclin1-regulated autophagy SFARI Gene Archive 5	**HIPPOCAMPUS** (Nobili et al., 2018) Reduction in the number of PV^+^ cells and PV protein levels (2–3 months)	**HIPPOCAMPUS** (Nobili et al., 2018) **Reduction of inhibitory drive on CA1 pyramidal neurons** • Reduced amplitude of dendritic eIPSCs • Reduced amplitude of Pvalb neuron-mediated perisomatic eIPSCs	Sociability and communication deficits (Nobili et al., 2018)
**Aspm**^**1−7**^ ***Aspm*** Abnormal spindle-like microcephaly associated	**HIPPOCAMPUS, TRN** (Garrett et al., 2020) Reduction in the number of PV^+^ cells (23 weeks)	n/a	Impaired short- and long-term object recognition memory (Garrett et al., 2020)
**PV-Lmo4**^**−/−**^ Lmo4 deletion in Pvalb neurons ***Lmo4*** LIM domain only	**ANTERIOR CINGULATE CORTEX** (Zhang et al., 2020a) No detectable loss of PV^+^ neurons (2–4 months)	**ANTERIOR CINGULATE CORTEX (ACC) L2/3** (Zhang et al., 2020a) **Altered properties of Pvalb neurons** • Increased membrane excitability and shortened latency to the first AP • Increased amplitude of spontaneous inhibitory inputs (mostly from Pvalb neurons) to the somata of ACC L2/3 pyramidal cells **Increased Pvalb neuron-mediated perisomatic feedforward inhibition**	Repetitive behaviors and deficits in social interaction (Zhang et al., 2020a)

It is unclear whether the previously reported reduction in the number of PV^+^ neurons is due to an actual decrease in Pvalb neuron count or PV downregulation because either of these would result in a smaller number of identifiable PV^+^ cells. In Pvalb neurons with a low PV expression level (~10–20 μM in hippocampus; Eggermann and Jonas, 2012)—but also in those with moderate expression (~100 μM in cortex and striatum)—a reduction in PV level could result in some Pvalb neurons falling below the threshold of detection by PV IHC, and they would thus be considered negative for PV. For example, in PV^+/−^ mice in which PV and *Pvalb* mRNA expression levels are reduced by ~50% in the forebrain (Filice et al., 2018) and cerebellum (Schwaller et al., 2004), the number of PV^+^ neurons quantified by stereological methods was decreased by ~25–30% in the SSC, mPFC, and striatum (Filice et al., 2016). As a rule of thumb, a decrease in PV protein expression by an average of ~50% results in 25–30% of Pvalb neurons expressing PV at a level below the threshold of detection by IHC. Importantly, unlike in humans, almost all prototypic Pvalb neurons [expressing γ-aminobutyric acid (GABA)] in the mouse brain are surrounded by PNNs, which can be detected based on the lectin-binding capacity of their glycan components by VVA or *Wisteria floribunda agglutinin* (WFA). In many brain regions, the overlap between PV^+^ and PNN^+^ neurons is >90% (Härtig et al., 1992). Therefore, the number of VVA^+^ cells is a realistic estimate for the number of Pvalb neurons in mice. In PV^+/−^ mice, the number of VVA^+^ cells was unaltered in all brain regions known to be associated with ASD—namely, SSC, mPFC, and striatum (Filice et al., 2016). Even in mice completely lacking PV (i.e., the number of PV^+^ cells was zero), the number of VVA^+^ neurons did not differ from that in WT mice. While there is a global reduction in PV expression in PV^+/−^ mice, in other ASD models, the decrease is brain region specific, yet the number of VAA^+^ neurons in these regions is unchanged. Reduced PV level and fewer PV^+^ neurons were observed in the SSC of Shank1^−/−^ (−30% PV^+^ cells) mice and in the striatum of Shank3B^−/−^ (−30%), Cntnap2^−/−^ (−30%), and *in utero* valproic acid (VPA)-treated (−15%) mice, suggesting that the striatum is a hotspot for the ASD-associated downregulation of PV. Notably, in the abovementioned mouse models, there are other brain regions with unaltered numbers of PV^+^ neurons (Schwaller, 2020), and the number of VVA^+^ cells is also unchanged. Moreover, in regions with a reduced number of PV^+^ neurons, *Pvalb* mRNA and PV protein expression levels were decreased by ~50% (or by ~30% in VPA mice). Thus, the Shank1^−/−^, Shank3B^−/−^, Cntnap2^−/−^, VPA, and PV^−/−^ ASD mouse models are characterized by PV downregulation and not the loss of Pvalb neurons. However, such information is unavailable for most of the ASD mouse models listed in Table 1, in which a decrease in the number/density of PV^+^ neurons has been reported in most cases. Nonetheless, such information can provide mechanistic insights as a decreased number or functional impairment of Pvalb neurons due to a reduction in PV is expected to alter the functioning of the neuronal network in distinct ways.

Irrespective of the cause of the decreased number of PV^+^ neurons, the mouse models listed in Table 1 exhibit changes at the level of electrophysiology and behavior. Notably, many of the genes listed in Table 1 are associated with ASD and linked to E/I imbalance (Table 1 in Lee et al., 2017). Processes/mechanisms contributing to physiologic E/I balance include the development of excitatory and inhibitory synapses, neurotransmission, and synaptic plasticity comprising homeostatic plasticity, activation of signaling pathways (e.g., excitation–transcription coupling), and modulation of intrinsic excitability (Nelson and Valakh, 2015; Lee et al., 2017). The few models in which such changes in electrophysiological properties are directly attributable to a decrease in PV level are described in detail in *Acute Reduction in PV Expression Induces ASD-Like Behavior in Juvenile Mice, While Promoting PV Expression in PV*^+/−^ *Mice Attenuates Social Behavioral Impairment*. Permanent changes in the firing properties of Pvalb neurons, their inputs, or their targets in ASD mouse models alter the steady-state equilibrium (i.e., E/I balance). It was initially proposed that an increase in the E/I ratio could be causally related to ASD (Rubenstein and Merzenich, 2003); however, it has since been demonstrated in many models that a homeostatic or maladaptive change in the E/I balance in either direction is a hallmark of ASD. The relative contribution of E/I imbalance to ASD can vary according to ASD risk/susceptibility gene mutation, brain region, specific features of synapses, and developmental time point. Even in mice harboring the same mutation, the E/I balance is differentially affected across neuron subpopulations. For example, mice carrying the neuroligin 3 mutation R451C (NL3^R451C^) show an increase in inhibitory synaptic transmission in SSC pyramidal cells—that is, increases in spontaneous inhibitory postsynaptic current (sIPSC) and evoked (e)IPSC frequency (Tabuchi et al., 2007) leading to a lower E/I ratio. However, in CA1 pyramidal neurons of the same mice, excitatory transmission mediated by N-methyl-d-aspartate (NMDA)- and α-amino-3-hydroxy-5-methyl-4-isoxazolepropionic acid (AMPA)-type glutamate receptors is increased. As the former shows greater involvement, the NMDA/AMPA ratio as well as E/I ratio are increased. The increase in NMDA/AMPA ratio does not occur in layer 2/3 pyramidal neurons of the SSC (Etherton et al., 2011). In the hippocampal CA1 pyramidal cells of Cntnap2^−/−^ mice, putative perisomatic input (likely via the axon initial segment, soma, and proximal dendrites) and eIPSC amplitude are decreased, while miniature excitatory postsynaptic current (mEPSC) frequency and amplitude are unchanged (Jurgensen and Castillo, 2015). However, there is a trend toward a lower NMDA/AMPA ratio in Cntnap2^−/−^ mice. On the contrary, the frequency (but not amplitude) of spontaneous action potential (AP)-driven IPSC is increased in mutant mice, whereas mIPSC is unchanged (both in frequency and amplitude). Strongly reduced layer 4 stimulation-evoked feedforward inhibition (i.e., eIPSC) and a smaller decrease in feedforward excitation (i.e., evoked EPSC) onto layer 2/3 SSC pyramidal cells results in a higher E/I ratio in several ASD mouse models including Cntnap2^−/−^ and Fmr1^y/−^ mice (Antoine et al., 2019). This only weakly affects spontaneous (basal) spiking in layer 2/3 pyramidal cells *in vitro*; however, the whisker-evoked firing rate of FSI (likely Pvalb neurons) is reduced in these two ASD models (Antoine et al., 2019). The above examples provide evidence that dysfunctional GABAergic transmission—often perisomatic inhibition by Pvalb basket cells—is a critical aspect of ASD. How the absence of PV is directly implicated in altered GABAergic transmission is discussed in *PV Affects Pvalb Neuron Targets and Inputs*.

### Acute Reduction in PV Expression Induces ASD-Like Behavior in Juvenile Mice, While Promoting PV Expression in PV^+/–^ Mice Attenuates Social Behavioral Impairment

Based on findings from ASD patients and ASD animal models discussed in *Decreased Number of PV*^+^ *Neurons in Human ASD Postmortem Brains: Pvalb Neuron Loss vs. PV* and *Downregulation* and *Animal NDD Models of ASD With Reduced PV Expression and/or Decreased Number/Altered Distribution of PV*^+^ *Neurons*, respectively, as well as our own *in vitro* studies (reviewed in Schwaller, 2020), we investigated the role of PV in the development of an ASD-like behavioral phenotype using genetically modified mice. Experimental approaches typically used to demonstrate causality between an experimental manipulation at the molecular or cellular level and higher-order brain function(s) (e.g., network activity or behavioral phenotype) are based on perturbation of the system. For instance, removing a component (e.g., a protein) hypothesized to be directly implicated in a behavior is predicted to lead to its appearance, whereas when the same component has lower abundance (e.g., in haploinsufficiency), upregulation to a normal level is expected to attenuate/alleviate the behavior (rescue experiment). There are several caveats to this concept. During the process of downregulation and/or reappearance of the investigated protein, additional adaptive, compensatory, or homeostatic changes may occur, likely over varying time scales and to different degrees. Nonetheless, the knockdown and rescue approaches have been successful in many instances, including in the field of ASD. Re-expression of Shank3 in a Shank3 conditional knock-in mouse model rescued social interaction deficits and attenuated repetitive grooming behavior in adult mice (Mei et al., 2016); early genetic restoration of WT Shank3 in Shank3^E13^ mutant mice rescued the same deficits as well as those in locomotion and rearing (Jaramillo et al., 2020). In MeCP2-overexpressing mice, reducing MeCP2 level (e.g., by antisense oligonucleotide treatment) reversed behavioral deficits associated with *MECP2* duplication syndrome (Sztainberg et al., 2015). Of note, null mutant *Shank3* and *Mecp2* mice are models for delayed and precocious circuit maturation during development, respectively, and especially for perisomatic Pvalb neuronal circuit function (Gogolla et al., 2014). Both mutants show impaired multisensory integration in the insular cortex, likely due to an E/I circuit imbalance skewed toward excitation (Shank3^−/−^) or inhibition (Mecp2^−/−^), respectively. This suggests that an optimal E/I circuit balance depends on precise Pvalb neuronal circuit function (Figure 7 in Gogolla et al., 2014). Alterations in the time course of network development/maturation—especially during critical periods of plasticity [postnatal days (PND) 5–15 in mice]—are thought to contribute to the appearance of ASD-like behavioral traits (Leblanc and Fagiolini, 2011).

We applied the perturbation (removal/readdition) approach to investigate the role of PV in the occurrence of ASD-like core symptoms in mice. We previously showed that PV^−/−^ mice constitutively lacking PV expression exhibited all of the core ASD symptoms—i.e., reduced reciprocal social interaction, impaired communication (ultrasonic vocalization), and repetitive and stereotyped behaviors (Wöhr et al., 2015); they also showed several ASD-associated comorbidities such as increased susceptibility to epilepsy (Schwaller et al., 2004), reduced pain sensitivity (Wöhr et al., 2015), slight impairment of motor coordination, and mild hyperactivity (measured as characteristic speed) (Farre-Castany et al., 2007). The same but weaker core ASD-like phenotype is seen in PV^+/−^ mice (Wöhr et al., 2015), in which the level of PV is about 50% lower than in WT mice (Schwaller et al., 2004; Filice et al., 2018). ASD-like behaviors in PV^−/−^ and PV^+/−^ mice have been consistently observed in the juvenile reciprocal social interaction assay and three-chamber assay evaluating direct social interaction and social preference, respectively. In the former, the most robust parameter distinguishing WT mice from those with reduced PV level (PV^+/−^ and PV^−/−^) was behavior following social behavior (Figure 3 legend and Figure 1D in Wöhr et al., 2015) and Figure 2C in Filice et al. (2018). In the three-chamber assay, the most relevant findings were related to sniff time—i.e., the time a mouse spent in close proximity (2 cm) to a caged stranger mouse – and not simply the time spent in the compartment with the stranger mouse; described in detail in the Supplementary Materials of Filice et al. (2018). To define the experimental conditions for the knockdown/rescue experiments, it is important to consider the developmental regulation of PV in Pvalb neurons. PV in mice is expressed starting from PND5 to PND7 (depending on the brain region), with peak expression occurring at approximately PND25–40 (Okaty et al., 2009). The developmental timeline of Pvalb neurons in the SSC (Figure 3 in Butt et al., 2017) and auditory cortex (Takesian et al., 2018) has been reported. An overview of cortical interneuron development can be found in Lim et al. (2018).

**Figure 2:**
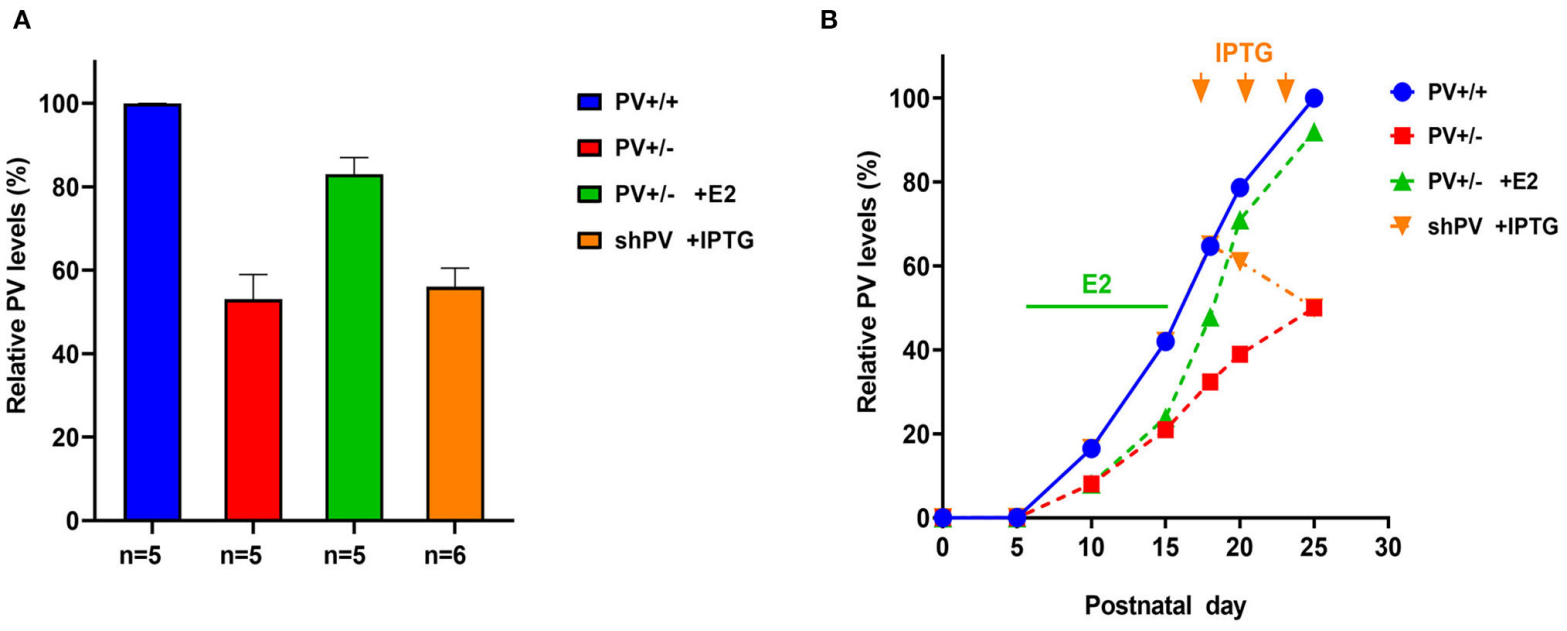
**(A)** Relative parvalbumin (PV) expression level determined by semi-quantitative western blot analysis in mouse forebrain extracts from PND25. Data for PV^+/+^ (defined as 100%), PV^+/−^, and PV^+/−^ mice treated with E2 are from Filice et al. (2018); data for isopropyl-β-d-thiogalactoside (IPTG)-treated shPV mice are from Filice et al. (2019). The number of mice is indicated; values represent mean ± SD. **(B)** Autism spectrum disorder (ASD)-like behavior shown in Figures 3, 4 was tested at PND25 in four conditions/genotypes: (1) wild-type (WT) mice; (2) constitutive PV^+/−^ mice; (3) PV^+/−^ rescue mice, in which PV expression was enhanced by E2 treatment; and (4) shPV22 (downregulation) mice, in which IPTG treatment at PND18, PND21, and PND24 reduced PV level to 50–60% of that in WT mice at PND25. PV expression profile (solid blue line) in WT (PV^+/+^) mice is based on data obtained from protein extracts of mouse cerebellum (Collin et al., 2005). In PV^+/−^ mice (red line), the level was assumed to be ~50% that of WT mice at all time points. Treatment of PV^+/−^ mice with E2 from PND5 to PND15 (green bar) results in a PV level that is ~90% of that in WT mice at PND25 (green line). Treatment of transgenic shPV22 mice with IPTG at PND18, PND21, and PND24 (orange arrows) decreases PV level to ~50% of that in WT mice at PND25 (orange line). As the precise course of PV expression in the three groups is not yet supported by experimental data, the lines are dashed. However, relative PV expression level at PND25 corresponds to the experimental data shown in **(A)**. As previously reported, IPTG treatment alone (at PND18, PND21, and PND24) had no effect on PV expression levels at PND25 as determined using the shTurboGFP control transgenic mouse line (Filice et al., 2019).

Quantitative data on PV expression levels during juvenile development in mice are available only for the cerebellum, which contains Purkinje cells and molecular layer interneurons (MLI) expressing PV (Figure 2B in Collin et al., 2005). These data were used to generate the blue curve for WT mice shown in Figure 2B. PV expression increases sharply starting at PND10 and reaches a plateau at PND20. The second postnatal week is also the start of the GABA switch from depolarizing to hyperpolarizing (observed in cultured hippocampal neurons) (Leonzino et al., 2016) that is essentially complete by PND12, as evidenced by the disappearance of giant depolarizing potentials (Ben-Ari et al., 1989; reviewed in Ben-Ari, 2002). Thus, the steep increase in PV expression begins at around the time when GABA action turns hyperpolarizing. The developmental course of PV expression may be different in other Pvalb neurons (in the cortex, hippocampus, and TRN). In PV^+/−^ mice, we assumed the same temporal course as in WT mice; however, PV level reaching only 50% of WT at PND25 (Filice et al., 2018) (red curve in Figure 2B). For the rescue experiment, PV^+/−^ mice were treated with 17-β-estradiol (E2) from PND5 to PND15, resulting in a PV expression level that was ~90% that of WT mice at PND25 (Filice et al., 2018). Hypothetical PV expression levels during the development of E2-treated mice are shown in Figure 2B (green curve). E2 has been shown to increase PV expression *in vitro* (Fujimoto et al., 2004) and in PV^+/−^ mice (Filice et al., 2018); in the latter, this effect is mediated by an as-yet unidentified estrogen-responsive element (ERE) in the mouse *Pvalb* promoter. As E2 most likely affects the expression of several genes with identified EREs, what are the evidences that E2-mediated upregulation of PV is a most likely cause for the attenuation of the ASD-like phenotype in E2-treated PV^+/−^ mice? Moreover, one has to take into account that E2 modulates social behavior in rats (Reilly et al., 2015) and more generally affects neural circuits via direct or indirect activation of multiple downstream signaling pathways (Marino et al., 2006). First, in PV^−/−^ mice, E2 had no obvious effect on PV expression or ASD-related behaviors (Filice et al., 2018). Second, although E2 treatment in WT mice does not increase global PV protein level, it is possible that PV protein is upregulated in some Pvalb neurons including those with low PV expression. Unexpectedly, E2 treatment provoked ASD-like behaviors in WT mice (e.g., decreased sociability and an increase in repetitive behavior). Possible reasons for this antisocial effect of E2 treatment in WT mice and additional arguments about the positive effect of E2 treatment selectively in PV^+/−^ mice are discussed in Filice et al. (2018). Stronger PV IHC signals indicative of elevated PV level were also observed in MeCP2^−/−^ ASD model mice (Patrizi et al., 2019), in accordance with the concept of optimal PV circuit function (Figure 7 in Gogolla et al., 2014). For the inverse experiment, we used the B6PV^Cre^-Tg(hPGK-eGFP/RNAi; Pvalb)^1Swal^ mouse line (abbreviated as shPV22 or shPV), which permits temporally controlled PV downregulation upon isopropyl-β-d-thiogalactoside (IPTG) administration (Filice et al., 2019). Induced expression of sh*Pvalb* from PND18 to PND25 decreased PV level to approximately 50–60% of the WT level (Filice et al., 2019), which was also comparable to the PV level in constitutive PV^+/−^ mice. The hypothesized course of PV expression level is shown in Figure 2B (orange curve). It should be noted that the only experimentally verified PV levels in the rescue and downregulation experiments are those measured at PND25 (Figure 2A)—that is, the time point of behavioral testing; the green and orange curves in Figure 2B depicting PV expression levels in the two models are thus estimates that are not yet supported by experimental data.

**Figure 3:**
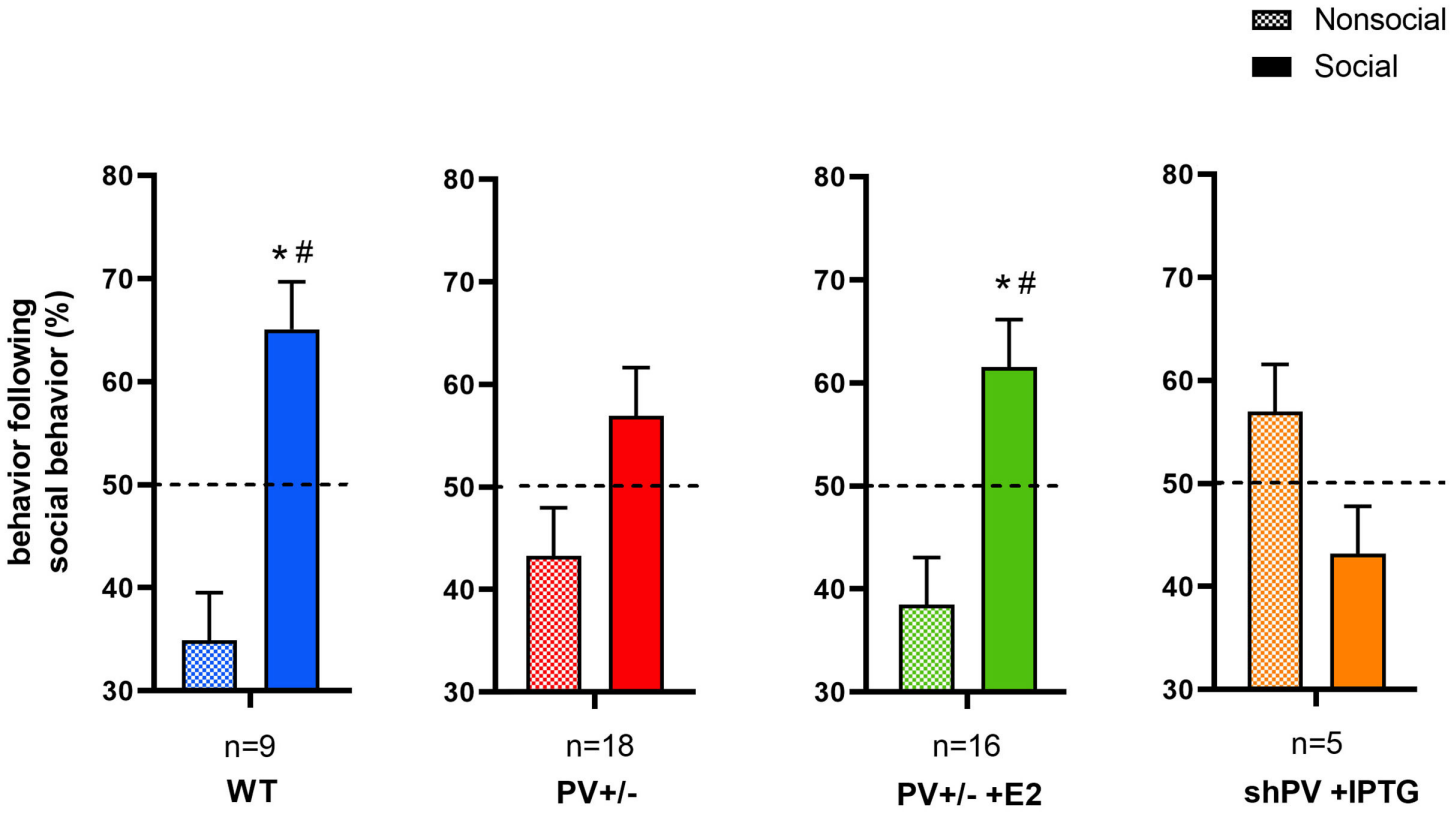
In the reciprocal interaction assay, the type and duration of each behavior is recorded, yielding ethograms for mouse pairs (Figure 1 in Wöhr et al., 2015). Behaviors classified as social include 1) facial and anogenital sniffing, 2) following, 3) social grooming, 4) pushing past, 5) crawling over-/under and 6) social inactive. Non-social behaviors include rearing, digging, and grooming. After each social-type interaction (independent of its duration) occurring during a 5 min observation period, the next interaction is either classified as social or non-social. From these data, the percentages of social and non-social interactions following a social interaction are calculated. In WT (blue bars) and E2-treated rescue PV^+/−^ (green bars) mouse pairs, a social interaction is likely followed by another social interaction. This preference is not observed in constitutive PV^+/−^ (red bars) or shPV mice, where parvalbumin (PV) is downregulated from PND18 to PND24 (orange bars); all mice were tested at PND25. It should be noted that the numbers shown (n) represent mouse pairs (i.e., the actual number of mice tested is 2n). Data for the first three groups are taken from Filice et al. (2018). Data for shPV mice is original. All other experimental details on breeding, testing, and data analysis can be found in Filice et al. (2018) and Wöhr et al. (2015) and briefly in Supplementary Materials. The dashed line indicates the 50% chance level. Filled and checkered bars represent social and non-social behaviors, respectively. *p < 0.05 vs. non-social; ^#^*p* < 0.05 vs. 50% chance level.

**Figure 4:**
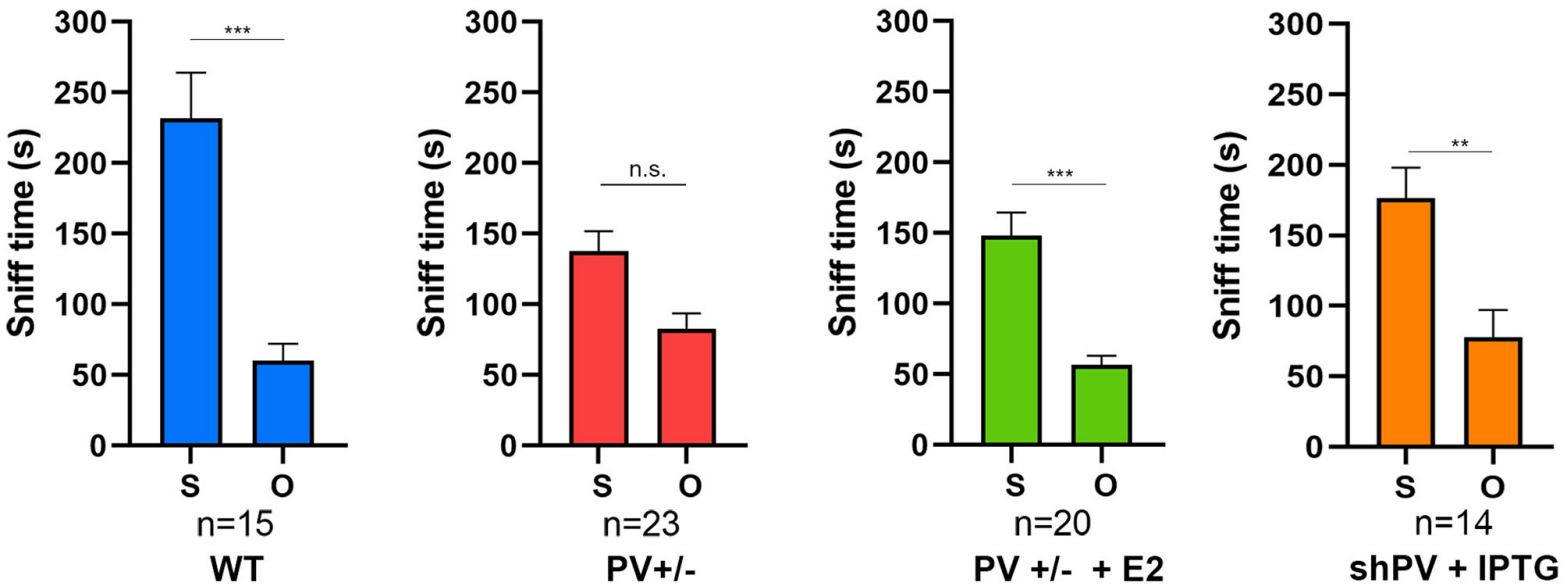
In the three-chamber assay testing social preference during a 10 min (600 s) observation period, the sniffing time is longer with a stranger mouse (S) than with an empty cage (O) in WT mice, an effect that is lost in constitutive PV^+/−^ mice (red bars). The preference for S is restored by upregulation of parvalbumin (PV) induced by E2 treatment (green bars). Data for the first three groups were previously reported (Filice et al., 2018). The preference for S is attenuated in shPV mice, in which PV is downregulated by isopropyl-β-d-thiogalactoside (IPTG) from PND18–24 (orange bars). Data for IPTG-treated shPV mice is original. All mice were tested at PND25. In the same shPV mice without IPTG treatment (sham-treated), the preference for S is similar to that of WT mice (data not shown). Data for the first three groups are from Filice et al. (2018). All other experimental details on breeding, testing, and data analysis can be found in Filice et al. (2018) and Wöhr et al. (2015). *Represents a significant preference for S over O. **p < 0.01, ***p < 0.001; n.s., not significant.

ASD-like behavior was tested at PND25 for four conditions/genotypes: (1) WT; (2) constitutive PV^+/−^; (3) PV^+/−^ rescue, in which PV expression was enhanced by E2 treatment; and (4) shPV22 (downregulation), in which PV expression declined starting from PND18 to 50–60% at PND25. Results from the first three groups have been published (Wöhr et al., 2015; Filice et al., 2018), while behavioral data from shPV22 mice have not yet been reported. WT mice showed a strong preference (>60%) for engaging in another social behavior after a previous one (Wöhr et al., 2015; Filice et al., 2018) (Figure 3); this preference was not observed in PV^+/−^ mice but was induced by increasing PV level through E2 treatment (Filice et al., 2018) and abolished after PV downregulation mediated by shPV22 starting from PND18. Almost identical results were observed in the three-chamber assay evaluating social preference: WT mice showed a stronger interest in the small cage with the stranger mouse than in the empty cage (object), as measured by sniff time (Figure 4). PV^+/−^ mice showed no preference for the stranger mouse, but this was restored by E2-mediated PV upregulation. Transient PV reduction induced by IPTG in shPV22 mice attenuated preference for the stranger mouse compared to WT mice [*p* = 0.024 in the first cohort (*n* = 6) and *p* = 0.179 in the second cohort (*n* = 4)]. However, when results from the two cohorts were combined, a preference (*p* = 0.0049) for the stranger mouse is maintained—as to a lesser extent (n.s.)—in constitutive PV^+/−^ mice (red and orange bars in Figure 4). In conclusion, induced downregulation of PV produces an ASD-like phenotype, while PV restoration in constitutive PV^+/−^ mice abrogates this phenotype. This strongly indicates that PV expression level is causally related to ASD-like behavior. In the next sections, mechanistic and anatomic evidence supporting this possibility are presented.

### PV Affects Pvalb Neuron Targets and Inputs

#### Biophysical Considerations

The function of an intracellular Ca^2+^ buffer (better termed as a Ca^2+^-signal modulator) like PV is simple in theory: binding Ca^2+^ ions at high intracellular Ca^2+^ concentration ([Ca^2+^]_i_) and releasing/unbinding Ca^2+^ when local Ca^2+^ concentration decreases. PV-specific biophysical properties include Ca^2+^ as well as Mg^2+^ affinity and kinetics. Only the most pertinent findings are discussed here; additional details can be found in Schwaller (2020). Data on the biophysical properties of PV were obtained from *in vitro* studies using purified recombinant PV. Rat PV contains two equivalent Ca^2+^/Mg^2+^-binding sites that are usually occupied by either of the two ions in a competitive manner, with dissociation constants (K_D_) of 11 nM and 41 μM, respectively (Eberhard and Erne, 1994). Modeling studies investigating the effect of PV on depolarization-induced increases in [Ca^2+^]_i_ in isolated chromaffin cells revealed physiologically relevant parameters including binding kinetics (Lee et al., 2000) and apparent Ca^2+^ affinities (i.e., K_D, Ca[app]_ values of 150–250 nM in an intracellular environment). Together with PV mobility determined in neuron somata [diffusion rate (D_PV_), ~40 μm^2^ s^−1^; Table 1 in Schwaller (2020)], these parameters should enable accurate prediction of the effect of a given concentration of PV on Ca^2+^ signals in a particular neuron if all other parameters relevant to Ca^2+^ signaling are known, such as the “on” mechanisms that lead to an increase in [Ca^2+^]_i_ as well as the “off” mechanisms that reduce [Ca^2+^]_i_ to basal levels (Berridge et al., 2003). However, such information is currently unavailable for essentially all proteins—for example, for a single voltage-gated Ca^2+^ channel Ca_V_2.1 (P/Q type; *CACNA1A*) or a plasma membrane calcium ATPase (e.g., PMCA1; *ATP2B1*) many splice variants exist, exhibiting distinct properties related to Ca^2+^ handling. All of these Ca^2+^ signaling components are present to varying degrees in different neuron subpopulations (some highly specific and others ubiquitous); even in supposedly homogeneous subpopulations such as cerebellar MLIs, PV expression levels vary considerably among individual neurons [average ~570 μM; range, 55–1,788 μM (Eggermann and Jonas, 2012)]. Moreover, average PV concentrations in Pvalb neuron populations in different brain regions are highly heterogeneous, ranging from 10 μM in the hippocampus to ~750 μM in TRN (Janickova et al., 2020). To further complicate matters, organelles previously assumed to be homogeneous such as mitochondria that are also involved in Ca^2+^ signaling/sequestration—especially in (pre)synaptic compartments (Devine and Kittler, 2018)—express a highly diverse set of proteins implicated in mitochondrial Ca^2+^ transport (Fecher et al., 2019), which is discussed in detail in *Effect of PV on Mitochondria, Oxidative Stress, and Pvalb Neuron Morphology: Link to NDDs*. Despite the complexity of the experimental data, certain global principles of neuronal Ca^2+^ signaling have been deduced.

The slow Ca^2+^-binding kinetics of PV generally exclude an effect on the fast-rising phase of Ca^2+^ transients, for instance those elicited by the opening of voltage- or receptor-operated Ca^2+^ channels such as Ca_V_2.1 (*CACNA1A*) or NMDA-type glutamate receptor (e.g., *GRIN1*). The same holds true for depolarization-evoked increases in [Ca^2+^]_i_ in PV-loaded chromaffin cells (Figure 7 in Lee et al., 2000). PV accelerates the initial rate of [Ca^2+^]_i_ decay, often transforming a monoexponential decay into a biexponential one with fast and slow decay components (τ_fast_ and τ_slow_, respectively) (Collin et al., 2005; Muller et al., 2007). This property of PV prevents/delays the gradual buildup of presynaptic [Ca^2+^]_i_ (e.g., within a train of APs) that subsequently affects short-term modulation of synaptic plasticity. This has been observed in a model system based on PV-loaded chromaffin cells (Lee et al., 2000). The following aspects of PV function can be understood from this model: (1) buildup of residual [Ca^2+^]_i_ is delayed but not prevented; (2) the effect of PV is dependent on the time interval between stimuli and is strongest at the time point when the [Ca^2+^]_i_ decay curve with or without PV shows the largest difference; (3) if stimuli are long enough [i.e., all PV molecules are loaded with Ca^2+^ (last part of the rising phase in Figure 7 in Lee et al., 2000)], the maximum amplitude of the Ca^2+^ signal is independent of PV; and (4) [Ca^2+^]_i_ decay following such a train is slowed/prolonged by Ca^2+^-loaded PV acting as a Ca^2+^ source (“on” mechanism). Evidence for a direct role of PV under these conditions is summarized below, although only a few selected examples are discussed; additional details can be found in the chapter on PV in Schwaller (2020).

#### Effect of PV on Synaptic Transmission

Higher residual Ca^2+^ in the absence of PV is the likely reason for increased paired-pulse facilitation (PPF) at the synapses between MLI and Purkinje cells (Caillard et al., 2000), hippocampal Pvalb neurons and CA1 pyramidal cells (Vreugdenhil et al., 2003), striatal Pvalb neurons and medium spiny neurons (MSN) (Orduz et al., 2013), and at the calyx of Held, a glutamatergic synapse (Muller et al., 2007). The last of these examples is an exception, as most Pvalb neurons are GABAergic; nonetheless, it demonstrates that the inhibitory effect of PV on PPF is independent of neurotransmitter type. The prevention (reduction) of facilitation is a hallmark of PV. The PV-mediated effect size on PPF depends on many parameters but most importantly on PV concentration, stimulation frequency [interspike interval (ISI)], number of spikes, and Ca^2+^ on/off kinetics in Pvalb neurons. The role of PV as a delayed Ca^2+^ source has been investigated in less detail, although delayed release at GABAergic synapses has been observed (Lu and Trussell, 2000; Kirischuk and Grantyn, 2003). At MLI–MLI synapses, an MLI firing in bursts (10 APs) induces long-lasting asynchronous release for up to 2,400 ms after the train; the duration is much shorter (400 ms) in PV^−/−^ MLIs (Collin et al., 2005). Interestingly, the IPSC integral of the delayed release in WT MLIs is greater than that of the signal generated during the AP train. Thus, the synaptic output of MLIs is random and almost constant during the interburst period; the mean signal intensity is essentially determined by the number of APs in the preceding burst. In summary, MLIs (and likely other Pvalb neurons, e.g., in the cortex) alternately adopt a phasic signaling mode during bursts and an integrating (tonic) signaling mode between bursts (Collin et al., 2005). PV has a different effect on sustained asynchronous release by layer 5 Pvalb neuron autapses in the SSC (Manseau et al., 2010). In the absence of PV, asynchronous release from the autaptic terminal is slightly but significantly increased; this decreases Pvalb neuron spike reliability and reduces the ability of pyramidal neurons to integrate incoming stimuli to produce precise firing. As each Pvalb neuron innervates multiple pyramidal neurons, asynchronous release from a single one may desynchronize a large portion of the local network and disrupt cortical information processing. While Pvalb autapses have been implicated in precise spike timing (required, for example, for synchronous oscillations) (Deleuze et al., 2019), asynchronous release reduces the spike precision and reliability of cortical neurons and thereby disrupts synchronous firing when the activity level surpasses a threshold value in order to prevent/decrease the generalized synchronous activity observed during epileptic discharges (Manseau et al., 2010). In the absence of PV (i.e., with increased asynchronous release), the onset of pentylenetetrazole-induced tonic–clonic seizures was found to be delayed in PV^−/−^ mice, although the intensity of the subsequent generalized seizures was higher (Schwaller et al., 2004).

Yet another effect of PV may contribute to the modulation of intracellular Ca^2+^ signaling, especially in Pvalb neurons with high, near-millimolar PV concentrations such as MLIs (Eggermann and Jonas, 2012) and TRN neurons (Janickova et al., 2020). In these neurons, the large pool of Mg^2+^-bound PV does not usually slow the effective Ca^2+^-binding rate but instead contributes to the regeneration of metal-free (apo) PV that acts as a fast Ca^2+^ buffer. Therefore, Mg^2+^ binding/unbinding may serve as a metabuffering (buffering of buffering) mechanism that maintains the concentration of apo-PV during rapid, repetitive activity in fast-spiking Pvalb neurons (Eggermann and Jonas, 2012; Hu et al., 2014). While proteins including K_V_3.1b are a prerequisite for the fast afterhyperpolarization that characterizes Pvalb neurons, extracellular matrix molecules of the mostly negatively charged PNN components contribute to their rapid firing. PNNs are thought to establish local molecular gradients of physiologically relevant ions such as Ca^2+^, K^+^, or Na^+^ around Pvalb neuron somata (Morawski et al., 2015). Accordingly, treatment with chondroitinase ABC—a bacterial enzyme that cleaves polysaccharide chains of PNN components—significantly (~50%) decreased Pvalb neuron firing frequency in cortical slices (Tewari et al., 2018).

PV influences spike reliability through asynchronous release and also during bursting activity. Besides the expected effect of increasing PPF at striatal Pvalb neuron–MSN synapses, the absence of PV leads to increased Pvalb neuron excitability and more regular spontaneous spiking within a train. Experimental (Orduz et al., 2013) and modeling (Bischop et al., 2012) data indicate that the likely cause of the latter is a change in the activation of small conductance (SK) Ca^2+^-dependent K^+^ channels. Thus, the presence of PV at the synapse induces arrhythmicity (i.e., increase in “jitter”) by modulating intrinsic oscillations via SK channel activation (Orduz et al., 2013). The triad of SK channels, voltage-dependent Ca^2+^ channels, and sarco(endoplasmic) reticulum Ca^2+^ ATPase (SERCA) pumps enables the generation of Ca^2+^ signaling-dependent oscillatory activity in the TRN (Cueni et al., 2008); in striatal Pvalb neurons and likely in TRN Pvalb neurons, PV is a fourth component that contributes to this process by increasing the irregularity of the oscillatory spiking pattern (Orduz et al., 2013). How the absence of PV affects TRN Pvalb neuron function *in vivo* is discussed in *Striatum and TRN as a Hub in NDDs: Role of Pvalb Neurons*.

Functional phenotypes observed in PV^−/−^ mice related to excitability, synaptic transmission, short-term modulation, firing properties, and network effects (e.g., oscillations) may represent the extreme end of decreased PV-induced changes because reduced but not complete loss of PV expression is usually observed in human NDDs and corresponding animal models. The functional consequences of a direct or indirect (i.e., secondary to NDD risk gene mutations) decrease in PV level are discussed here. A small hairpin (sh)RNA was used to assess the role of PV in cortical circuits of adolescent rats (PND34–38) (Caballero et al., 2020). PV level in the PFC was decreased by ~25% at PND65, resembling the NDD rodent phenotype of lower PV expression at an older age (>PND60). In layer 5 pyramidal cells, the frequency of sIPSCs was reduced and PV deficiency was observed, which increased facilitation (i.e., a higher PPF ratio at an ISI of 50 ms). As sEPSC frequency was unchanged, the reduction in PV led to an increase in the E/I ratio in pyramidal cells. On the contrary, the frequency of sEPSCs was decreased in fast-spiking cortical Pvalb neurons. Hippocampal-evoked local field potential (LFP) responses in the PFC are affected by a lower PV level in a frequency-dependent manner: at 20 and 40 Hz, LFP facilitation occurs while control rats show LFP depression. In summary, higher PV expression is required for refinement of prefrontal GABAergic function, while its absence results in immature afferent processing and a hypofunctional state (Caballero et al., 2020).

Results from the above-described study cannot be directly compared to experiments carried out in PV^+/−^ mice. Unlike in PV-shRNA rats, no data are available from PV^+/−^ mice on LFPs in the PFC *in vivo* or IPSCs mediated by fast-spiking (Pvalb) neurons and EPSCs determined *ex vivo*. Moreover, behavioral data from PV-shRNA rats are sparse. In the trace fear conditioning and extinction test, acquisition of a cue-mediated fear response was unaffected by a reduction in PV level, while fear extinction was prolonged. Compared to controls, PV-shRNA rats showed increased freezing times lasting until the last trial of cue presentation (Caballero et al., 2020). Similar findings were obtained in the reward-based T-maze reversal learning assay carried out in PV^−/−^ mice to assess behavioral inflexibility: while the initial learning phase was identical for WT and PV^−/−^ mice, a large proportion of the latter exhibited a clear deficit in the ability to reverse their behavior in order to receive the reward (Wöhr et al., 2015). It will be interesting to determine whether PV-shRNA rats also exhibit repetitive and stereotyped ASD-like behaviors. Additionally, electrophysiological recordings of the PFC in PV^+/−^ mice may reveal similarities between the two models in terms of neuronal firing properties and behavior.

#### Alterations in Synaptic Transmission in NDD Mouse Models Suggest PV Involvement

Mice lacking the ASD-associated gene *LMO4* (encoding an endogenous inhibitor of PTP1B phosphatase) specifically in Pvalb neurons [PV-*Lmo4* knockout (KO) mice] show increased Pvalb neuron-mediated perisomatic feedforward inhibition (likely by basket cells) onto pyramidal cells (Zhang et al., 2020a), as well as increased excitability of Pvalb neurons in the dorsal anterior cingulate cortex (dACC). Spontaneous inhibitory inputs (sIPSC amplitude) mostly from Pvalb neurons onto dACC layer 2/3 pyramidal cells are increased while excitatory inputs are less affected, resulting in a lower E/I ratio. Optogenetically induced activation of Pvalb neurons was shown to increase PPF. Differences between WT and KO mice were greatest at an ISI of around 20–50 ms, while short-term depression of IPSCs in a train (e.g., at 20 Hz) was reduced in KO mice. The broadened AP signal in PV-*Lmo4* KO mice may be attributable to a reduction in delayed rectifying potassium conductance (by K_V_1.2 or K_V_3) (Zhang et al., 2020a). Given the close association (coexpression) of PV and K_V_3.1b in most cortical Pvalb neurons (Chow et al., 1999), a decrease in K_V_3.1b is a potential mechanism for AP broadening. It is worth noting that, at the behavioral level, PV-*Lmo4*KO mice display typical ASD core symptoms of reduced social interaction and repetitive, stereotyped behaviors (Table 1).

As discussed above and shown in Table 1, PV expression is altered in several genetic and “environmental” mouse ASD models (*Animal NDD Models of ASD With Reduced PV Expression and/or Decreased Number/Altered Distribution of PV*^+^ *Neurons*). Changes in proteins involved in synaptic transmission have been reported in these mice and are briefly summarized here. The level of *Kcnc1* transcript encoding the K_v_3.1b channel necessary for maintaining the fast-spiking phenotype of Pvalb neurons was decreased slightly in PV^−/−^ mouse and significantly (−40%) in VPA mouse forebrain lysates (including neocortex, thalamus, and pallidus) (Lauber et al., 2016). The latter mice are characterized by reduced PV expression in the striatum (Lauber et al., 2016). In the cortex, K_v_3.1b shows near-perfect overlap with PV (99% of all PV^+^ neurons are K_v_3.1b^+^ and vice versa) (Du et al., 1996; Chow et al., 1999; Lien and Jonas, 2003). The expression and function of hyperpolarization-activated cyclic nucleotide-gated (HCN) channels responsible for I_h_ currents are important for regulating resting membrane potential, input resistance, dendritic integration, synaptic transmission, and neuronal excitability (Biel et al., 2009; Benarroch, 2013). These channels are altered in ASD mouse models, often in those with decreased PV expression. This is the case for Shank3B^−/−^ mice expressing a lower level of PV in striatal Pvalb neurons (Filice et al., 2016). Cultures of developing hippocampal neurons derived from these mice show increased input resistance and excitability as well as drastically attenuated I_h_ currents and reduced expression of Hcn4 protein (Yi et al., 2016). Reduced *Hcn4* mRNA and PV levels are also seen in the striatum of Cntnap2^−/−^ mice (Lauber et al., 2018). Moreover, *Hcn1* mRNA expression in the striatum was decreased slightly in PV^−/−^ and significantly in Shank3B^−/−^ mice at PND25 (Lauber et al., 2016). On the contrary, *Hcn1* mRNA and HCN1 protein levels were elevated (by ~40%) in the forebrain of PND25 VPA mice that showed no difference in forebrain PV expression relative to the control (Lauber et al., 2016). Thus, brain region- and gene-specific (*Gad1, Pvalb, Kcnc1, Hcn1-4*, and *Kcnn1–4*) alterations may be responsible for (maladaptive) homeostatic mechanisms and are observed in several ASD models, mostly those with decreased PV level; some of these are likely cell autonomous and linked to Pvalb neuron hypofunction (e.g., *Gad1, Pvalb*, and *Kcnc1*), as are the downstream consequences of reduced (absent) PV expression such as increases in the number of mitochondria and dendritic branching (as discussed in *Effect of PV on Mitochondria, Oxidative Stress, and Pvalb Neuron Morphology: Link to NDDs*). Altered expression of HCN family members *Hcn1–4* or potassium calcium-activated channel subfamily N members 1–4 (SK channels) that are not specifically expressed in or even absent from Pvalb neurons reflect a Pvalb neuronal circuit phenotype caused by PV downregulation-mediated functional changes in Pvalb neurons. However, the putative changes (e.g., alterations in neuronal activity of one neuron subpopulation leading to parallel or hierarchical changes in others) have not been fully elucidated.

Adding yet another level of complexity, the absence of PV in striatal PV neurons has a presumed retrograde effect on the cortical glutamatergic inputs onto Pvalb neurons. In the time window (10–50 ms) when Pvalb neuron–MSN synapses show stronger facilitation in the absence of PV, PPF of EPSCs between cortical neurons and striatal Pvalb neurons—mostly mediated by AMPA receptors—is significantly reduced (Wöhr et al., 2015). The same effect is observed in PV^+/−^ mice, in which a ~50% reduction in PV level was sufficient to influence PPF at this corticostriatal synapse. Further experiments have suggested that changes in short-term plasticity at the cortical neuron–FSI synapse involve a presynaptic adaptation mechanism, possibly resulting from homeostatic plasticity in the cortical neuron caused by the absence/reduction in PV in the postsynaptic striatal Pvalb neuron. However, the lack of a direct link (synapse) between Pvalb neurons and their cortical inputs indicate a retrograde or circuit (network) effect. In summary, the removal/decrease in PV in Pvalb neurons has complex effects on intracellular Ca^2+^ signaling and synaptic transmission even without considering the changes induced at the morphological level (discussed in *Effect of PV on Mitochondria, Oxidative Stress, and Pvalb Neuron Morphology: Link to NDDs*).

### Striatum and TRN as a Hub in NDDs: Role of Pvalb Neurons

Several brain regions and associated circuits have been investigated in the context of NDDs including the basal ganglia and thalamus, and especially the TRN. The basal ganglia are involved in motor learning, habit formation, and stimulus processing to generate appropriate responses; they are also implicated in mood, motivation, and decision making (goal-directed action). These processes involve the cortico–basal ganglia–thalamocortical loop in which cortical pyramidal cells project to the striatum, with striatal neurons giving rise to direct and indirect pathways. In the former, the neurons project to the internal segment of the globus pallidus, which sends efferents to the thalamus; projections from the thalamus to the cortex close the loop. In the indirect pathway, a fiber bundle from the striatum reaches the external segment of the external globus pallidus, which forms synapses with neurons of the subthalamic nucleus (STN). The STN projects to the thalamus and from there back to the cortex. The term “disclosed loop” has been used to describe a closed circuit that is open to outside influence at the initial stage of cortical input (Figure 8 in Shipp, 2017). Patients with a damaged striatum often show autistic traits (Damasio and Maurer, 1978; Maurer and Damasio, 1982) and stereotyped movements. In male mice, dorsal striatum-specific damage (by coablation of ~40–50% Pvalb neurons and large cholinergic interneurons) results in spontaneous stereotypy and deficits in social interaction (Rapanelli et al., 2017). Restricted and repetitive behaviors are also linked to alterations in striatal and thalamic circuits (Farmer et al., 2013). Functional brain imaging studies have revealed the involvement of corticostriatal–thalamic circuits in ASD (Fuccillo, 2016), and morphological studies of the thalamus and striatum indicate differences such as a larger volume (Schuetze et al., 2016) and surface area and greater rostrocaudal variation in the shape of the thalamus in ASD patients compared to control subjects (Schuetze et al., 2019). ASD rodent models with striatal and thalamic alterations have been developed to investigate the mechanisms underlying such dysfunctions. Details on the striatal impairment in these mutant mice can be found in Table 1 of Li and Pozzo-Miller (2019).

Mutations in several ASD-associated genes affect corticostriatal connectivity (reviewed in Fuccillo, 2016; Li and Pozzo-Miller, 2019) or general striatal structure or function as observed in human ASD patients (Langen et al., 2007; Estes et al., 2011) and multiple ASD mouse models such as Fmr1^−/−^ (Centonze et al., 2008), Shank3^−/−^ (Peca et al., 2011), Cntnap2^−/−^ (Penagarikano et al., 2011), and Cntnap4^−/−^ (Karayannis et al., 2014). Another possible feature of corticostriatal dysfunction in ASD is abnormalities in Pvalb neurons, including decreased PV expression in striatal Pvalb neurons likely caused by mutations in ASD risk genes such as *CNTNAP2* and *SHANK3* (Table 1).

Although Pvalb neurons account for just 1% of striatal neurons (Tepper et al., 2010), they are responsible for ~10% of all striatal activity (Duhne et al., 2020). Striatal Pvalb neurons receive projections from different cortical regions (mainly primary motor cortex and primary SSC) as well as inhibitory input from TRN (Klug et al., 2018), which is often described as the “guardian of the gateway” (Crick, 1984), and selectively modulates the thalamocortical network, playing a critical role in sensorimotor and cognitive functions, sleep, and consciousness (Crick, 1984; Schmitt et al., 2017). It is unsurprising that abnormalities in the thalamocortical circuitry are associated with some of the sensory perceptual deficits typical of ASD; thus, TRN dysfunction is considered as a prototypical circuit endophenotype in NDDs (Krol et al., 2018). NDD risk genes associated with schizophrenia (*CACNA1I* and *GRM3*) and ASD (*CHD2* and *PTCHD1*) are strongly expressed in TRN, and as they are also thought to be linked to Pvalb neurons, results obtained in the mutant mice are discussed here. TRN neurons comprise two (mainly non-overlapping) populations characterized by expression of either PV (~60% of neurons) or SST (~40%) (Clemente-Perez et al., 2017; Steullet et al., 2017a). The cell types differ in terms of intrinsic membrane excitability and low-threshold Ca^2+^ current (*I*_*T*_) mediated by Ca_V_3.2 and Ca_V_3.3 (Talley et al., 1999). Peak *I*_*T*_ density is higher in Pvalb neurons, suggesting that SST neurons have fewer and/or more dendritically located T-type calcium channels (Clemente-Perez et al., 2017). Hyperpolarization of TRN neurons leads to low-threshold Ca^2+^ spikes in the form of rebound bursts, each crowned by a series of APs. The maximal number of rebound bursts, number of APs after the first burst, and intraburst frequency are higher in Pvalb neurons than in SST cells (Clemente-Perez et al., 2017). Ca_V_3.3 channels are required for TRN cell bursting and synchronized rhythmicity in the thalamic circuit. Absence of Ca_V_3.3 channels (*Cacna1i*) in Ca_V_3.3^−/−^ mice decreases *I*_*T*_ by ~80% and almost completely abolishes the bursting properties of TRN neurons (that likely express Pvalb) (Astori et al., 2011). A lack of Ca_V_3.3 channels also reduces apamin-sensitive currents mediated by SK2 channels, indicating that Ca_V_3.3/SK2 channel interactions are required for the proper functioning of the latter in TRN neurons (Cueni et al., 2008). Interestingly, KO mouse models of ASD and schizophrenia have shown similar findings with respect to TRN neuron function both *in vivo* and in slice cultures.

*In vivo* recordings of TRN Pvalb neurons in anesthetized PV^−/−^ mice have revealed altered proportions of neurons with specific firing properties: medium-bursting (type II) neurons were more abundant than the long-bursting type (III), indicating a decrease in rebound bursting in the absence of PV. Additionally, the ISI within a burst was longer, while the number of spikes was unaffected (Alberi et al., 2013). These changes are likely associated with an altered distribution of Ca_V_3.2 channels mediating *I*_*T*_. Ca_V_3.2 channels are more abundant at active axosomatic synapses of PV^−/−^ TRN neurons as compared to WT TRN neurons, suggesting that the differential localization of Ca_V_3.2 affects bursting dynamics. Notably, the distribution of Ca_V_3.3 channels is similar in the two genotypes. TRN neurons express apamin-sensitive SK channels (Cueni et al., 2008), and the absence of PV is therefore presumed to have a similar effect on TRN neuron SK currents as in the striatum (Orduz et al., 2013). A cross-correlation analysis of neurons simultaneously recorded with the same electrode tip showed that ~30% of neuron pairs tended to fire synchronously independent of PV expression. Hence, PV deficiency does not affect the functional connectivity between TRN neurons that are also coupled by chemical synapses (Zhang et al., 2020b), as is the case in cortical Pvalb neurons (Galarreta and Hestrin, 1999), but affects the distribution of Ca_V_3.2 channels and dynamics of burst discharges in TRN cells, possibly by modulating SK channel activity. Other KO mouse models of Ca^2+^ signaling components (e.g., the schizophrenia risk gene *Disc1*) show a strong effect on the function of TRN neurons, which most likely involves SK channels (Delevich et al., 2020). In mice, patch-domain containing protein 1 (PTCHD1) is expressed almost exclusively in TRN neurons at PND0 and is still prominently expressed at PND15. Ptchd1^Y/−^ mice with TRN neuron-specific deletion of the ASD- and intellectual disability-associated *Ptchd1* gene showed attention deficit and hyperactivity along with a 50% reduction in SK currents, which were rescued by a pharmacological treatment that restored SK channel function (Wells et al., 2016). Importantly, at all investigated membrane potentials of Ptchd1^Y/−^ TRN neurons in cultured slices, the number of rebound bursts was smaller than in WT mice, whereas no difference was observed in *I*_*T*_. Thus, the reduced bursting activity of Ptchd1^Y/−^ TRN neurons is likely the result of decreased SK currents. Moreover, free [Ca^2+^]_i_ is significantly lower in Ptchd1^Y/−^ TRN neurons, providing further evidence of altered intracellular Ca^2+^ handling.

### Effect of PV on Mitochondria, Oxidative Stress, and Pvalb Neuron Morphology: Link to NDDs

The two major roles of mitochondria are the generation of ATP via oxidative phosphorylation and Ca^2+^ buffering. In neurons, these two processes occur not only globally but also locally—for example, in presynapses (reviewed in Devine and Kittler, 2018). The electrophysiological properties of Pvalb neurons require both fast presynaptic Ca^2+^ handling and sufficient energy production to sustain high-frequency firing. PV is a component of the Ca^2+^ signaling toolkit involved in the regulation of intracellular [Ca^2+^]_i_ (Berridge et al., 2003). A high firing rate of cortical GABAergic interneurons including Pvalb neurons is observed *in vivo*, which is more physiologically relevant than the fast-firing properties of Pvalb neurons that were mostly measured *in vitro*. Two-photon *in vivo* calcium imaging in the SSC of mice has revealed that GABAergic interneurons have higher activity than excitatory neurons, measured as event rate using the Ca^2+^ indicator GCaMP6 expressed in either neuron population (Chen et al., 2020). PV acting as a slow-onset Ca^2+^ buffer mostly serves as a “Ca^2+^ off” mechanism that reduces [Ca^2+^]_i_ after an initial increase in conjunction with two other essential “Ca^2+^ off” components—namely, SERCA pumps and PMCAs—that require ATP for this function. How the absence or partial downregulation of PV affects a cell expressing PV under physiological conditions remains an open question. A Ca^2+^ overlay blot with protein extracts of forebrain and cerebellum did not show upregulation of EF-hand Ca^2+^-binding proteins similar to PV in the brain of PV^−/−^ mice (Schmidt et al., 2003). On the other hand, fast-twitch muscles of PV^−/−^ mice are more resistant to fatigue as a result of increased fractional volume (density) of mitochondria (Chen et al., 2001). This inverse (antagonistic) regulation (i.e., PV downregulation and overexpression increasing and decreasing mitochondrial volume, respectively) occurs in all systems investigated to date including tissues/cells endogenously expressing PV such as fast-twitch muscle cells, kidney epithelial cells, and Pvalb neurons (Schwaller, 2020). The same mechanism exists in cells overexpressing PV including neurons of Thy-PV mice (Van Den Bosch et al., 2002; Maetzler et al., 2004), C2C12 muscle cells (Ducreux et al., 2012), MDCK kidney epithelial cells (Henzi and Schwaller, 2015), and CG4 oligodendrocyte-like cells (Lichvarova et al., 2019). Mitochondria are functionally similar to PV in terms of shaping intracellular Ca^2+^ transients: neither greatly affects the rising phase because of their slow-onset Ca^2+^-buffering/Ca^2+^-uptake properties but increases the rate of [Ca^2+^]_i_ decay, thus shortening Ca^2+^ transients, and both have anti-facilitating effects at presynaptic terminals—i.e., they accelerate recovery from synaptic depression (e.g., in the calyx of Held; Kim et al., 2005), thereby participating in the clearance of presynaptic Ca^2+^ and modulating neurotransmitter release (Billups and Forsythe, 2002). According to the notion of activity-dependent homeostatic changes/adaptations (Berridge et al., 2003), mitochondria are in the best position (with respect to Ca^2+^ signal modulation) to compensate for a loss/decrease in PV. Moreover, while SERCA pumps and PMCAs are not obviously upregulated in PV-deficient cells, they are presumed to contribute more extensively to Ca^2+^ removal in the absence of PV (although this has yet to be demonstrated). Thus, PV-deficient Pvalb neurons likely need to produce more ATP to accommodate the increased activity of Ca^2+^ extrusion systems. Ca^2+^ uptake by mitochondria is energetically costly and can only be maintained by increasing ATP production (Palmieri and Persico, 2010). Conversely, Ca^2+^ buffering by PV is an energy-saving process, as the small conformational changes in PV induced by Ca^2+^ binding/Ca^2+^ release require little energy and are independent of ATP levels.

Mitochondria were previously assumed to be homogeneous in terms of composition and function but are now known to be highly diverse within different organs (Mootha et al., 2003) and even in different cells of the brain, as demonstrated in cerebellar granule cells, Purkinje cells, and astrocytes (Fecher et al., 2019). This is also true of cell components involved in Ca^2+^ signaling, which show cell-type specific regulation (Fecher et al., 2019). Several experimental models with reduced PV expression show not only an increased mitochondrial volume but also elevated levels of mitochondrial proteins directly related to Ca^2+^ uptake. In epithelial cells of the distal convoluted tubule in mouse kidney, several genes implicated in mitochondrial Ca^2+^ uptake and generation of mitochondrial membrane potential including mitochondrial calcium uptake 1 *(Micu1*), mitochondrial calcium uniporter regulator 1 (*Mcur1*), and mitochondrial cytochrome c oxidase subunit 1 (*mt-Co1*) are upregulated in PV^−/−^ mice (Henzi and Schwaller, 2015). Levels of *COXI* and positive regulators of mitochondria biogenesis (*Ppargc1a, Nrf1*, and *Tfam*) are higher in PV-negative CG4 cells than in those overexpressing PV (Lichvarova et al., 2019). However, no differences are observed in the expression of mitochondrial genes directly linked to ATP synthesis such as F1-ATPase subunit (*Atp5Ff1b*) (reviewed in Schwaller, 2020), further suggesting that the increases in mitochondrial volume and protein expression likely enhance the slow Ca^2+^-buffering capacity conferred by PV in WT cells. Additionally, transcript levels of mitochondrial uncoupler protein UCP2 (*Ucp2*) are elevated in control (PV^−^) CG4 cells (similar to the condition in PV^−/−^ mice) compared to PV-expressing CG4 cells (mimicking the condition in WT Pvalb neurons) (L. Janickova, unpublished). This is in line with RNA-seq results from cortical samples of ASD patients demonstrating that *UCP2* is among the most strongly upregulated transcripts (Parikshak et al., 2016; Schwede et al., 2018). Consistent with findings from PV^−/−^ mice, transcript levels of several genes related to synaptic transmission and mitochondria are altered in ASD (Schwede et al., 2018), and expression-weighted cell type enrichment analysis of three mouse ASD and schizophrenia models revealed significant upregulation of mitochondrial genes in fast-firing inhibitory (presumably Pvalb) neurons, while mitochondrial protein levels showed a global downregulation in the cortex (Gordon et al., 2019).

The increase in the density of mitochondria is not homogeneous in the soma of Pvalb neurons and is mostly observed in the region adjacent to the plasma membrane in Purkinje cells (Chen et al., 2006) as well as in striatal and TRN Pvalb neurons (Janickova and Schwaller, 2020). Previously, it was unclear whether this is an on/off or precisely regulated mechanism. Analyses of nine different Pvalb neuron populations from five different brain regions (hippocampus, cortex, striatum, cerebellum, and TRN) selected based on moderate-to-strong PV staining revealed a strong positive correlation between the magnitude of the increase in mitochondrial density and estimated PV concentrations in Pvalb neurons of WT mice—i.e., a higher PV concentration was associated with a greater PV deficiency-induced increase in mitochondrial density (but not in the length of individual mitochondria). Such an increase exists not only in PV^−/−^ Pvalb neuron somata but also in the dendrites (Janickova et al., 2020), confirming an earlier finding that mitochondrial density was higher in control CG4 (PV^−^) cells than in those overexpressing PV (Lichvarova et al., 2019). In particular, more mitochondria are present at the end of elongating processes and near the growth cone in control CG4 cells, which likely promotes process branching similar to what is observed in PV^−/−^ striatal and TRN Pvalb neurons (Janickova et al., 2020). Whether increased dendrite length and branching in the absence of PV results in local hyperconnectivity as observed in several ASD mouse models (Table 1 in Janickova et al., 2020) remains to be determined. More detailed information on mitochondria and branching can be found in the Discussion of Janickova et al. (2020).

The fast-firing property of Pvalb neurons is associated with their high energy demand (Kann et al., 2014; Kann et al., 2014); accordingly, Pvalb neurons contain significantly more mitochondria in their somata, dendrites, and axons than any other interneuron subtype or pyramidal cells. This makes Pvalb neurons especially vulnerable to metabolic stress and/or excessive formation of reactive oxygen and nitrogen species (ROS and NOS, respectively) (Kann, 2016). ROS production increases with age in Pvalb neurons of WT mice and is highly correlated with PV concentration but not mitochondrial density, which is similar (~8%) in all investigated Pvalb neurons (Janickova and Schwaller, 2020); that is, between the ages of 1 and 6 months, a higher PV concentration is associated with an age-dependent increase in ROS production. Importantly, in the absence of PV, ROS production is significantly higher in mice older than 1 month and the relative increase is approximately proportional to the PV concentration in WT Pvalb neurons (Janickova and Schwaller, 2020). An elevation in ROS levels causing an imbalance in redox homeostasis has been suggested as a pathophysiological mechanism of ASD (Tang et al., 2013; Pangrazzi et al., 2020). Increased ROS levels have also been observed in postmortem brain tissue of children with ASD (Siddiqui et al., 2016).

Increased mitochondrial ROS production and Pvalb neuron impairment (possibly linked to decreased PV level) have been causally linked to schizophrenia etiology and pathology (reviewed in Hardingham and Do, 2016; Steullet et al., 2016, 2017b). In Gclm^−/−^ mice—a schizophrenia mouse model deficient in the glutamate cysteine ligase modifier subunit, the rate-limiting enzyme for glutathione biosynthesis (Cabungcal et al., 2019)—the number of PV^+^/PNN^+^ neurons is decreased. This may be due to Pvalb neuron loss or a decrease in PV expression and altered molecular composition of PNNs, possibly as a result of neuron immaturity (Steullet et al., 2017a). In brain slices from Gclm^−/−^ mice, most TRN neurons were shown to fire in a tonic mode at a membrane potential of −70 mV, whereas ~80% neurons in WT mice exhibit bursting behavior. At more negative membrane potentials (−80 and −90 mV), no differences in bursting behavior existed between genotypes. While membrane potential-dependent differences in firing behavior of TRN neurons are correlated with decreased PV level in Gclm^−/−^ TRN neurons, a causal relationship between these events has yet to be established. Notably, a decrease in the number of PV^+^ neurons in the TRN has been reported in other ASD models showing behavioral phenotypes such as the Engrailed-2 KO mice (En2^−/−^) and abnormal spindle-like microcephaly-associated gene (Aspm1–7) mutants (Garrett et al., 2020; Pirone et al., 2020). Whether this is due to PV downregulation—and the electrophysiological consequences thereof—remains unclear.

Brain-development-dependent mitochondrial alterations (dysfunction) may trigger a cascade of events that leads to NDDs such as ASD or schizophrenia (Tang et al., 2013; Cabungcal et al., 2019). However, at least in 1 month-old PV^−/−^ mice showing no increase in ROS production (Janickova and Schwaller, 2020) but exhibiting robust ASD-like behaviors, excessive oxidative stress can be excluded as the main contributor to the ASD phenotype. The onset of oxidative-stress-induced Pvalb neuron dysfunction may occur at a later age and result in a schizophrenia-like rather than an ASD-like phenotype, although this is speculative and must be tested in longitudinal studies using different NDD mouse models. Supporting the oxidative stress hypothesis of NDD, inhibiting oxidative stress in schizophrenia patients by treatment with the antioxidant N-acetyl-cysteine (NAC) significantly improved neurocognition and alleviated positive clinical symptoms (Klauser et al., 2018; Retsa et al., 2018). However, in young individuals with ASD, NAC treatment for 12 weeks boosted glutathione production but did not improve social interaction deficits (Wink et al., 2016). Meanwhile, symptoms of irritability were attenuated in children with ASD who were treated with NAC (Hardan et al., 2012). Additional studies are required to determine whether NAC can reduce ASD core symptoms.

## Conclusions and Outlook

We have formulated the Parvalbumin Hypothesis of ASD based on experimental data (new and previously published) (Figure 1). This hypothesis pertaining to the etiology of ASD (and possibly schizophrenia) provides a framework for future research and allows the formulation of predictions that can be tested in human ASD postmortem brain samples or using the many existing animal models of ASD. This hypothesis does not hold true for all ASD cases and may even be restricted to a small number of cases with known genetic mutations or environmental perturbations in which downregulation of PV has been reported. The validation of a hub consisting of PV-expressing neurons in ASD is expected to facilitate the development of therapeutic strategies that restore brain function(s) in ASD and possibly other NDDs.

## Data Availability Statement

The raw data of novel experiments described in the article supporting the conclusions of this article will be made available by the authors, without undue reservation.

## Ethics Statement

The animal study was reviewed and approved by the Cantonal Veterinary Office (Canton of Fribourg, Switzerland). Experiments were performed according to institutional guidelines of the present Swiss law and the European Communities Council Directive of 24 November 1986 (86/609/EEC). The authorization number for housing of mice is H-04.2012-Fr and for the experiments 2016_37_FR and 2019_24_FR.

## Author Contributions

FF, LJ, TH, AB, and BS contributed to data collection (literature research). FF, TH, and AB carried out the experiments and performed data analysis. BS conceived the concept of the article. All authors were involved in writing the paper and read and approved the final manuscript.

## Conflict of Interest

The authors declare that the research was conducted in the absence of any commercial or financial relationships that could be construed as a potential conflict of interest.
