# Supplementary material for: The Parvalbumin Hypothesis of Autism Spectrum Disorder
Source: Front Cell Neurosci. 2020 Dec 18;14:577525. doi: 10.3389/fncel.2020.577525 (PMC7775315; doi:10.3389/fncel.2020.577525)
Supplement: Supplementary file 1 [file Data_Sheet_1.PDF]

Supplemental Methods and Materials

Testing of behavior in shPV mice: reciprocal interaction assay and 3-chamber assay

Animal housing and treatment

B6PVCre-Tg(hPGK-eGFP/Rnai; Pvalb)^{1Swal} mice (Filice et al., 2018), hereafter shPV mice, were housed in a temperature-controlled animal facility (24°C, 12:12 h light/dark cycle), at the University of Fribourg, Switzerland and fed *ad libitum* (for more information about this mouse line, see (Filice et al., 2019)). Only male animals were used in this study. Mice were randomly assigned to one of two experimental groups. Animals in the first group received IPTG intraperitoneally at a dose of 1 mg in 200 µl saline solution at PND18, 21, 24 ± 1 day, while mice belonging to the second group received a sham intraperitoneal injection (saline solution) at the same time points. Mice were weaned at PND21 and tested at PND25 ± 1 for reciprocal social interaction and at PND26 ± 1 for social approach in the 3-chamber assay, accordingly to previously established protocols (Wöhr et al., 2015;Filice et al., 2018).

Reciprocal social interaction assay

Mice were socially isolated for 24 h prior to testing. Pairs of juvenile mice (non-littermates, same treatment) were allowed to socially interact at PND25 ± 1 for 5 min after one mouse of the pair had been habituated to the test environment for 1 min. Behavior was recorded using a video camera placed 30 cm away from the cage. Social interactions were scored by an experienced observer blinded to the experimental conditions using the Observer XT 10.0 software (Noldus Information Technology, Wageningen, The Netherlands). For the parameters scored as 'social behaviors', see Figure 3 legend and (Wöhr et al., 2015). All social behaviors were analyzed for frequency of occurrence and duration.

3-chamber assay

Sociability in shPV mice (IPTG- or sham-treated) was determined by the 3-chamber social approach task (Yang et al., 2011). For the detailed protocol, see (Filice et al., 2018). After a 10-min habituation period with the test mouse free to move in all the empty compartments of the chamber, the same mouse was then briefly confined to the center chamber. A novel object (empty wire cup) was placed in a distant corner of one of the side chambers, while a wire cup identical to the one used as object, but containing a novel mouse, was placed in the distant corner of the other side chamber. The novel mouse was previously habituated (10 min) to the wire cup. The 'stranger' stimulus mice were also shPV mice (neither treated with IPTG nor saline) of the same sex and age as the test subjects. The locations of the novel

object and the novel mouse were alternated between the left and right chambers across test subjects to avoid a side preference bias. After both stimuli were positioned, the two side doors of the middle compartment were simultaneously lifted and the subject mouse had access to all three compartments for 10 min. The time spent in each compartment and entries into each one, as well as the time spent exploring the novel mouse or the empty cup were manually scored by an observer blinded to the mouse genotype/treatment using two stopwatches. Exploration of an enclosed mouse or of the empty wire cup was scored positive, if the test mouse was oriented with the head towards the cup within a 2-cm distance between the head of the mouse and the cup, or when climbing on top of the cup.

All experiments were performed with permission of the local animal care committee (Canton of Fribourg, Switzerland) and according to the present Swiss law and the European Communities Council Directive of 24 November 1986 (86/609/EEC).

Statistical analysis

For analysis of reciprocal social interaction, paired t-tests were used to compare the likelihood of the occurrence of a social behavior in response to a social behavior, and one-sample t-tests for comparisons with chance levels. Two-way ANOVA for repeated measurements with the within-subject factor preference (mouse *vs*. object) and the between-subject factors treatment (saline *vs*. IPTG) were used to analyze the social approach behavior in the 3-chamber assay. ANOVA was followed by paired Student's t-tests when appropriate.

References

Filice, F., Blum, W., Lauber, E., and Schwaller, B. (2019). Inducible and reversible silencing of the Pvalb gene in mice: An in vitro and in vivo study. *Eur J Neurosci* 50**,** 2694-2706.

Filice, F., Lauber, E., Vörckel, K.J., Wöhr, M., and Schwaller, B. (2018). 17-beta estradiol increases parvalbumin levels in Pvalb heterozygous mice and attenuates behavioral phenotypes with relevance to autism core symptoms. *Mol Autism* 9**,** 15.

Wöhr, M., Orduz, D., Gregory, P., Moreno, H., Khan, U., Vörckel, K.J., Wolfer, D.P., Welzl, H., Gall, D., Schiffmann, S.N., and Schwaller, B. (2015). Lack of parvalbumin in mice leads to behavioral deficits relevant to all human autism core symptoms and related neural morphofunctional abnormalities. *Transl Psychiatry* 5**,** e525.

Yang, M., Silverman, J.L., and Crawley, J.N. (2011). Automated three-chambered social approach task for mice. *Curr Protoc Neurosci* Chapter 8**,** Unit 8 26.
